# Extracellular vesicles of U937 macrophage cell line infected with DENV-2 induce activation in endothelial cells EA.hy926

**DOI:** 10.1371/journal.pone.0227030

**Published:** 2020-01-07

**Authors:** Myriam Lucia Velandia-Romero, María Angélica Calderón-Peláez, Arturo Balbás-Tepedino, Ricaurte Alejandro Márquez-Ortiz, L. Johana Madroñero, Alfonso Barreto Prieto, Jaime E. Castellanos

**Affiliations:** 1 Grupo de Virología, Vicerrectoría de Investigaciones, Universidad El Bosque, Bogotá, Colombia; 2 Laboratorio Genética Molecular Bacteriana, Vicerrectoría de Investigaciones, Universidad El Bosque, Bogotá, Colombia; 3 Grupo de Inmunobiología y Biología Celular, Facultad de Ciencias, Pontificia Universidad Javeriana, Bogotá, Colombia; Utah State University, UNITED STATES

## Abstract

Endothelial activation and alteration during dengue virus (DENV) infection are multifactorial events; however, the role of extracellular vesicles (EVs) in these phenomena is not known. In the present study, we characterized the EVs released by DENV-2 infected U937 macrophage cell line and evaluated the changes in the physiology and integrity of the EA.hy926 endothelial cells exposed to them. U937 macrophages were infected, supernatants were collected, and EVs were purified and characterized. Then, polarized endothelial EA.hy926 cells were exposed to the EVs for 24 h, and the transendothelial electrical resistance (TEER), monolayer permeability, and the expression of tight junction and adhesion proteins and cytokines were evaluated. The isolated EVs from infected macrophages corresponded to exosomes and apoptotic bodies, which contained the viral NS3 protein and different miRs, among other products. Exposure of EA.hy926 cells to EVs induced an increase in TEER, as well as changes in the expression of VE-cadherin and ICAM in addition leads to an increase in TNF-α, IP-10, IL-10, RANTES, and MCP-1 secretion. These results suggest that the EVs of infected macrophages transport proteins and miR that induce early changes in the physiology of the endothelium, leading to its activation and eliciting a defense program against damage during first stages of the disease, even in the absence of the virus.

## Introduction

Eukaryotic cells secrete extracellular vesicles (EVs) into the bodily fluids, such as blood, saliva, urine, or breast milk [1]. These EVs are spherical particles surrounded by a phospholipid bilayer containing proteins or nucleic acids that control a broad range of biological functions when they are sensed by a receptor cell [2]. The composition of EVs varies depending on the type of cell from which they arise, or the cellular status thereof [3, 4]. They may be classified according to size, source, protein composition, mRNA and miR content, biological function, or biogenesis. Regarding their biogenesis, EVs may be divided into three groups: exosomes, microvesicles, and apoptotic bodies (ABs) [5].

Exosomes are 30–100 nm diameter structures with a flotation density of 1.13 to 1.19 g/mL [6] and a characteristic cup shape when observed under a transmission electron microscope (TEM) [7]. These vesicles are surrounded by a cholesterol, sphingomyelin, and ceramide-rich phospholipid membrane [8] and contain transport and fusion proteins (GTPases, flotillin, and annexin), tetraspanins (CD9, CD63, CD81, and CD82), heat shock proteins (Hsp70 and Hsp90), intraluminal vesicle biogenesis-related proteins (Alix and TSG-101), cytoskeleton proteins (actin and tubulin), signaling proteins (kinases, 14-3-3 protein, and trimeric G proteins), and some enzymes (GAPDH, acetylcholinesterase, and elongation factors). Additionally, exosomes may also contain small RNA (miR), mRNA, and other non-coding RNAs [8].

However, during apoptosis, fragmented cells produce another kind of vesicles known as apoptotic bodies, ABs [9]. These bodies are heterogeneous, with a broad range of sizes (50–5000 nm) and a flotation density varying between 1.16 and 1.28 g/mL. Like exosomes and microvesicles, ABs are involved in intercellular communication processes through the biomolecules they transport, such as RNAs, proteins, and lipids [10–12]. However, these vesicles are unique because they also contain organelle debris and DNA fragments [13]. Due to its ability to bind to exposed phosphatidylserine in the outer membrane, annexin is the classic AB marker [3, 13], thrombospondin, the complement component C3b and some histones are also detected [3, 11]. There are two kinds of ABs: one originated from the plasmatic membrane (containing DNA and histones), and the other from the endoplasmic reticulum (exposing immature glycoepitopes) [5]. Independently of their source, ABs participates in some pathologic conditions, such as inflammation, autoimmunity, oncogenesis, and viral infections [12] since some viruses induce apoptosis in infected cells [14]. Viruses such as human immunodeficiency virus (HIV) [15], rotavirus [16], and Japanese encephalitis virus (JEV) [17], kidnap the cellular biogenesis machinery and secretion of EVs and utilize them for virion assembly and release. Other viruses such as Epstein–Barr virus (EBV) [18] and human cytomegalovirus–hCMV- [19] use EVs to evade the immune response and to transport viral regulatory factors, like proteins, RNAs, and miRs, from an infected cell to the surrounding cells. Together, these strategies allow the virus to improve its spreading capacity and impact upon host cells [20].

Regarding cellular response modulation, it has been reported that EVs from HIV-infected astrocytes can induce a steady expression of cytokines in CD4+ T lymphocytes, exacerbating the depletion and death of this type of cell [21], or causing them to release EVs that activate other uninfected CD4+ T lymphocytes [22]. Also, miRs transported in EVs from EBV-infected cells prepare and favor viral replication in epithelial cells and induce ICAM-1 expression [23]. Similarly, EVs isolated from cerebrospinal fluid from JEV-infected patients exhibit high concentrations of miRs that can regulate the MAPK pathway and the expression of transforming growth factor-β (TGF-β) in the target cells [17]. Another virus that kidnaps and modulates cellular response through EVs is the flavivirus hepatitis C (HCV), transporting proteins like NS5A [24], capsid proteins, or viral RNA [25] in exosomes from infected hepatocytes that can infect, replicate, and modulate cellular response in target cells [26, 27]. These strategies allow HCV to evade immune responses and increase its spreading and persistence, even in the presence of neutralizing antibodies [28, 29].

Interestingly, in the context of DENV (another flavivirus), despite being one of the most damaging arboviruses affecting individuals worldwide [30], little is known about its ability to use EVs as a competitive advantage. The DENV possesses a single-strand RNA that encodes a unique polyprotein that is co-translationally processed to generate three structural (C, PrM, and E) and seven nonstructural (NS1, NS2A, NSB, NS3, NS4A, NS4B, and NS5) proteins [31, 32]. Development of severe dengue fever (previously known as dengue hemorrhagic fever) is associated with changes in the physiology of endothelial cells (ECs), which are not only susceptible to viral infection but are also a target of the immune response elicited during infection [33, 34]. This alteration has been associated with circulation of inflammatory mediators and other molecules affecting EC physiology [35]. However, to date, it is unknown if EVs derived from DENV-infected cells participate in endothelial activation and dysfunction. Therefore, in the present study, we characterize the EVs released by macrophages of the U937 cell line infected with DENV-2 and we evaluate their effect on the EA.hy926 endothelial cell line.

## Materials and methods

### Ethics statement

The main project “Assessment of exosomes produced by macrophages and its participation during endothelial activation and Dengue Virus serotype-2 spread” was approved by the Ethics institutional committee of Universidad El Bosque, File #011–2014.

### Cell culture, DENV-2 infection, and cell viability

Human adherent U937 (ATCC® CRL-1593.2^™^) macrophages were previously established by treatment with phorbol myristate acetate that induces a state of differentiation with morphological and biochemical characteristics like those of primary macrophages, being an optimal cellular model to study the infection by different intracellular pathogens [36]. These cells were maintained in 75 cm^2^ flasks under non-pyrogenic conditions using DMEM (Lonza, Switzerland) supplemented with 10% fetal bovine serum (FBS, Gibco) and penicillin-streptomycin (PS) (100 U/mL-100 μg/mL (Gibco)). Upon reaching 70% confluence, the cells were exposed to DENV-2 (Colombian isolated and grown in C6/36 cells) MOI: 0.2 for 1 h with gentle rolling (35 rpm).

Mock-infected cells were incubated during 1 h with non-infected C6/36 mosquito cell lysate; then, macrophages were washed with PBS, and cells were maintained for an additional 96–120 h post-infection (p.i.) with defined medium BIO-MPM-1 (Biological Ind) supplemented with 2 mM glutamine (Thermo Fisher Scientific). The supernatants from 10 culture flasks were processed with a total of approximately 1.2 × 10^7^ cells. To evaluate cell viability, infected and mock-infected cells were detached at 96 or 120 h p.i., seeded into a 96-well plate, and incubated with Calcein-AM 2.5 mM (Thermo Fisher Scientific) prepared in phenol red-free medium, and fluorescence was measured using a TECAN Infinite 200 fluorometer.

### Immunofluorescence for DENV and counting

Five thousand U937 cells were seeded on 10 μg/mL poly-L-lysine-treated round glass coverslips in 24-well plates and infected or treated with mock-inoculum for 96 h. Cells were fixed with 4% paraformaldehyde (PFA) (Carlo Erba), washed with 50 mM ammonium chloride, and permeabilized with 0.3% Triton X-100 for 30 min. Unspecific binding sites were blocked with 10% goat serum (Gibco) for 30 min, and then the anti-flavivirus monoclonal antibody (MAB 8744 Millipore) was added to detect the viral E protein. After extended PBS washing, biotinylated goat anti-mouse IgG was added (BA-9200 Vector); followed by the Alexa 594-coupled streptavidin (Thermo Fisher Scientific) for 30 min, then the cells were washed with PBS before marking the nuclei counterstaining with Hoechst dye. Coverslips were mounted using Vectashield mounting media^®^ (Vector) and monolayers observed in a Zeiss Axio Imager.A2 (Germany) using the X-cite 120Q fluorescence system and Zen 2012 software. Cells were then counted using ImageJ software; eight random fields were analyzed to count infected and non-infected cells to obtain infection percentages. Three independent cultures were evaluated with two replicates per condition.

### Extracellular vesicle isolation

Infected and mock U937 macrophage cell supernatants were collected at 120 h p.i. and centrifuged 10 min at 300 × *g* to pellet cell debris. The supernatants were centrifuged again at 2,000 × *g* for 20 min at 4°C, and the resulting supernatants were filtered through 0.22 μm nitrocellulose membranes (Merck Millipore). These eluates were concentrated using Amicon Ultra-15 MWCO 100K centrifugal concentrators (Merck Millipore) at 4,000 × *g* for 20 min at 4°C. The concentrated preparation was precipitated via ultracentrifugation (UC) at 100,000 × *g* for 60 min at 4°C using an Optima MAX-TL centrifuge (TLS-55 rotor -k Factor 100.2- Beckman Coulter), and the pellet was resuspended in 100 μL PBS and stored at −80°C until use. Pellets from DENV-infected macrophages was named *P (+)* and that from non-infected cells was named *P (-)*.

### Protein concentration and acetylcholinesterase activity (AChE)

The enzyme activity was determined in the pellets using a Red Amplex^®^ Acetylcholine / Acetylcholinesterase Assay Kit (A12217) (Thermo Fisher Scientific Rockford IL, USA) following the manufacturer's instructions. In brief, a dilution 1:10 of *P (+)* and *P (-)* pellets were resuspended in the reaction buffer; then, the working solution was added (Red Amplex^®^ reagent 400 μM, horseradish peroxidase 2 U/mL, choline oxidase 0.2 U/mL, and acetylcholine 100 μM), and the reaction mixture was incubated at room temperature for 30 min. Fluorescence was measured using a TECAN Infinite fluorometer with 530 nm (Ex) and 590 nm (Em) wavelengths. The results were interpolated from a standard curve of AChE made simultaneously (100 to 500 mU). Amounts of acetylcholinesterase were normalized to the protein concentration of each sample obtained via bicinchoninic acid assay.

### Extracellular vesicles flotation density determination

Pellets *P (+)* and *P (-)*, were resuspended in 2.5 M sucrose prepared in HEPES/NaOH 20 mM buffer, dispensed on a 2.5 to 0.5 M sucrose gradient, and separated using UC for 15 h at 100,000 × *g* (Beckman L8-55M, SW41 rotor, k factor 256.6) as previously reported [16]. Fractions (1 mL) were collected, and density was determined using a refractometer (ausJENA). Fractions were then diluted in 100 μL of PBS, pelleted 1 h at 100,000 × *g* (Optima MAX-TL, T LA rotor 100.3 factor k 60.6) and resuspended in 6X Laemmli buffer for Western blot (WB) analysis.

### SDS-PAGE and Western blot

Pellets *P (+) and P (-)* (20 μg/μL) and were separated on 10–12% polyacrylamide gels and silver stained or transferred to a PVDF membrane (Thermo Fisher Scientific) for 3 h and were then incubated with blocking solution (Tris–HCl pH 7.5, 0.5% Tween-20, 2–5% of non-fat dried milk or BSA). As a positive control, we evaluated whole cells lysates of infected and uninfected macrophages U937 obtained with RIPA buffer (Nonidet P40 1%; Deoxicolate 0,5%; SDS 1% in PBS). Protein concentration was determined with the BCA protocol and 15 μg/μL was loaded in the polyacrylamide gels and transferred to PVDF membrane as previously described.

The membranes were incubated with primary antibodies overnight at room temperature to detect different cell or viral proteins. Antibodies against ESCRT complex markers such Alix (1:1000, Cell Signaling), TSG-101 (1:1000, Thermo Fisher Scientific), exosome markers such as CD63 (ab68418 ABCAM), and AB marker Histone-3 (H3, 1:2000, Cell Signaling) were used as primary antibodies. To detect DENV proteins, rat antibodies against NS3 and NS5 were used as previously reported [37]. Finally, after extended washing, membranes were incubated for 1 h with respective HRP-coupled secondary antibodies: IgG anti-mouse (1:1000, ECL Amersham^™^, product #NA931); IgG anti-rabbit (1:2000, Thermo Fisher Scientific, product #31460); or IgG anti-rat (Thermo Fischer Scientific, product #31470). Reactions were visualized using SuperSignal^®^ West Pico Chemiluminescent substrate (Thermo Fisher Scientific) and images captured using a ChemiDoc^™^ Imaging System (BIO-RAD).

### Mass spectrometry proteomics (nano LC-MS/MS)

Pellets *P (+) and P (-)* (30 μg / μL) were resuspended in 6X Laemmli buffer. Then, each sample was loaded on a SDS-PAGE gel and run for 5 min at 200 V (to concentrate the proteins in a single band). The gel was then stained with colloidal Coomassie (Coomassie G-250 0.1%, 10% ammonium sulfate and 3% orthophosphoric acid), and the stained area was excised and sent for analysis via liquid chromatography coupled with tandem mass spectrometry (LC-MS/MS) using services offered by Alphalyse A/S (Odense, Denmark). According to the company, the samples were reduced and alkylated with iodoacetamide (carbamidomethylan), then digested with trypsin. The resulting peptides were concentrated by lyophilization with Speed -Vac and resuspended for injection in a Dionex nano-LC system and MS-MS analysis in a Bruker Maxis Impact Q-TOF instrument. The MS-MS spectra were used for searching on the Mascot database, then, the data was searched in the internal protein database downloaded from UniProt containing all the known non-redundant protein sequences.

### Transmission electron microscopy (TEM)

An aliquot of the pellets *P (+)* was fixed with 4% paraformaldehyde and 2% glutaraldehyde solution and deposited on 200 mesh nickel grids treated with Formvar. Samples were stained with lead citrate and uranyl acetate and observed using a Zeiss EM109 electron microscope (Jena, Germany).

### Extracellular vesicles immunoprecipitation

The pellets *P (+)* and *P (-)* were incubated with an Exo-Flow 96 Exosome immunoprecipitation kit (Exo-Flow 32ACD63; System Biosciences) following the manufacturer's instructions. Briefly, 50 μL of each pellet were incubated overnight at room temperature with magnetic beads coated with CD63 antibody, then placed on a magnetic plate for bead immobilization, recovered, and stored at −80°C until use. The immunoprecipitates from infected U937 cells were called *EVs (+)* and mock treated U937 cells *EVs (-)* and were processed by SDS-PAGE and WB to detect the proteins Alix, NS3 (of DENV) and H3.

### Evaluation of extracellular vesicles infectivity

To determine the infectious capacity of EVs, 15,000 LLC-MK2 cells (ATCC® CCL-7^™^) were incubated with 20 μl of *EVs (-)*, *EVs (+)* or *EVs (+)* pretreated with neutralizing antibody against DENV D1 - 4G2 -4-15 (4G2) (Merck Millipore; 1.5 μg / mL for 1 hour at 37°C) *(*called *EVs (+)*_*4G2*_). We also use as positive control, cells incubated with DENV-2 (MOI: 0.2) in the presence or absence of the neutralizing antibody, cells incubated with the supernatant free of beads (BFS) or *EVs (+)* irradiated UV *(*called *EVs (+)*_*UV*_) and the non-exposed cells were evaluated. After 2 hours of incubation at 37°C, the coating medium (1.5% carboxymethylcellulose) was added; at 72 hpi, the layers were fixed with 4% PFA.

**Fig 1:**
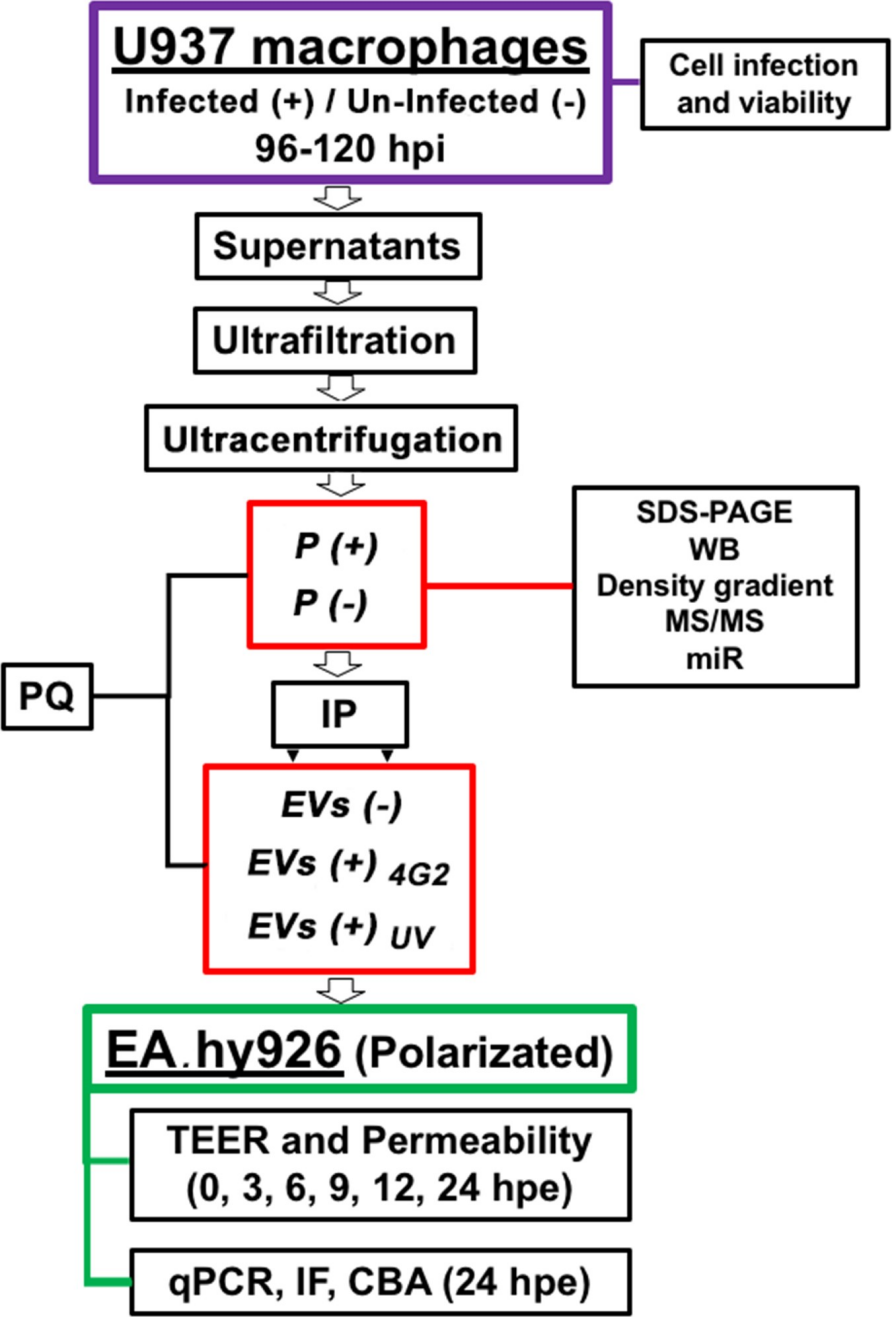
Flow diagram that summarizes the experimental procedure. Macrophages U937 cells were infected (+) with DENV-2 at a MOI: 0.2, or remained uninfected (-). After 96 or 120 hours post infection (h p.i.) the percentage of viability and cell infection was evaluated. Then, supernatants were collected, ultrafiltrated and ultra-centrifuged. The pellet of the infected *(P (+))* or uninfected macrophages *(P (-))* were characterized. Another part was processed by immunoprecipitation (IP) with magnetic beads coupled with a CD-63 antibody. The obtained *EVs* (either summited to the IP process or not) were evaluated by plaque assay (PQ). EVs from the infected macrophages (*EVs (+))* were irradiated with UV *(EVs (+)*_*UV*_*)* or treated with the neutralizing antibody 4G2 *(EVs (+)*_*4G2*_*)*. Then, the pellets or EVs were inoculated on endothelial cells EA.hy926 and the TEER and permeability was evaluated at different times. At 24 hours post-exposure (h.p.e.) RNA was extracted from the EA.hy926, another part of the cells was processed by immunofluorescence (IF) and the supernatants by CBA. As controls, non-exposed cells (NEC) or cells infected (IC) with DENV-2 MOI: 0.2 were evaluated.

The Focus Formation Units (FFU) were detected and counted after immunoperoxidase processing. For this the LLC-MK2 cells were permeabilized, the non-specific sites and the activity of the endogenous peroxidases were blocked and incubated with the flavivirus monoclonal antibody (MAB 8744 Millipore), secondary biotinylated antibody and streptavidin peroxidase were used and revealed with DAB and H_2_O_2_ as we described previously [38]. The FFU were determined with an inverted microscope AxioVert 40 (Zeiss, Jena, Germany) using the formula: M × DF × 50, where M is the average number of foci counted; DF: dilution factor; 50: correction factor to express the titer in FFU / ml (1000 / inoculum volume) [39]. This part of the methodology is summarized in Fig 1.

### Endothelial cell polarization and assessment of barrier alteration

EA.hy926 endothelial cells (ATCC^®^ CRL-2922^™^) were maintained under non-pyrogenic conditions using DMEM with sodium pyruvate (Biowest) supplemented with 10% FBS and 1% PS. At 80% confluence, cells were trypsinized and 60,000 cells were seeded in the apical domain (upper chamber) of a Corning^®^ Transwell^®^ polyester membrane cell culture insert (Sigma-Aldrich) previously treated with Collagen IV (Sigma-Aldrich) and Fibronectin (Invitrogen) and cultured at 37°C and 5% CO_2_ during 12 h. At this point and using a voltmeter Millicell ERS-2 (Millipore), initial TEER values were measured (time 0). After the latter, cells were exposed to pellets (*P (+)* and *P (-)*) and vesicles (*EVs (-) and (+)*, *EVs (+)*_*UV*_ and *EVs (+)*_*4G2*_) using a dilution of 1:10 (vesicles:medium). Regularly, TEER measurements were taken during the next 12 h (in 3 h intervals), and then at 24 h. Additionally, to assess permeability, cultures were supplemented with 2.3 mg/mL dextran blue 2000 (DB) and supernatants from the basal domain (lower chamber) of the cell culture inserts were collected at the same times and evaluated in a spectrophotometer at 630 nm [40]. Moreover, cells seeded in transwell® membranes were fixed with 4% PFA and then processed through IF to determine the expression of VCAM (Santa Cruz SC-8304), ICAM (Santa Cruz SC-8439), PECAM (Santa Cruz SC- 1506) and E-Selectin (Santa Cruz SC-14011), following the protocol previously described to detect the E protein of the virus.

After 24 hours post-exposure (h p.e.), total RNA from polarized cells was extracted using Trizol® (Invitrogen). Relative expression of VCAM, ICAM, and PECAM transcripts was determined using specific primers and normalized against β-actin messenger, as previously described [38]. Additionally, supernatants from the apical domain of the cell culture inserts were used to quantify cytokines (TNF-α, IP-10, IL-10, IL-12, MCP-1, IFN-α, RANTES, IL-12p70, IL-8, IL-6, and IFN-γ) with the LEGENDplex Human Th Cytokine Panel (BioLegend^®^) after 24 h p.e. (methodology also summarized in Fig 1).

### Small RNAs characterization of EVs

To detect and characterize small RNA (miR), the *P (+)* and *P (-)* were treated with 100 μg/mL of RNase A (Thermo Fisher Scientific) for 1 h, pelleted again via UC at 100,000 × *g*, and resuspended in the lysis buffer of mirVana miRNA isolation kit (Thermo Fisher Scientific). Then, following the manufacturer's instructions acid phenol-chloroform solution was added, and phases were separated by centrifugation. The recovered aqueous phase was washed with ethanol, placed into the cartridge provided with the isolation kit, and centrifuged at 10,000 × *g*. Membranes were extensively washed, and RNA eluted with the appropriate buffer and stored at −80°C. Verification of RNA concentration and quality was performed on a capillary electrophoresis RNA Bioanalyzer using the Small RNA chip system with the TruSeq^®^ SmallRNA Library Prep Kit (Illumina Inc.), and multiplexed cDNA libraries were prepared. Size distribution (140–160 bases, including index and sequence adapters) and concentration (>1.5 ng/μL) of libraries were verified on the Bioanalyzer using the DNA 1000Chip. Final libraries were single-end sequenced on one lane for 51 cycles using the Hiseq2500 platform (Illumina Inc.). FASTQ files containing more than 40 million reads per sample were processed to remove index, adaptor sequences, and low-quality bases using the Cutadapt and Trimmomatic tools. Then, we filtered out reads shorter than 12 and longer than 32 nucleotides using Cutadapt software, and overrepresented sequences (a sequence having a number of appearances in the library representing over 0.1% of the total reads in the FASTQ file) were filtered out and inspected for miRNAs in the miRBase (www.mirbase.org) database.

### miR target genes prediction network regulation construction and GO enrichment analyses

Target genes of each expressed miR were retrieved from four databases: mirbase [41], mirtarbase [42], TargetScan (TS) [43] and TransmiR [44] using the CyTargetLinker [45] app and its interactions and regulatory network were constructed in cytoscape v. 3.7.

The predicted target genes for each expressed miRNA in *P (+)*, *P (-)* and in both conditions were submitted to Gene Ontology (GO) enrichment analysis using GOrilla tool [46]. GO terms for Ranked gene lists in each condition were considered as enriched terms when its p-value was ≤ 0.001 according to the minimum hypergeometric (mHG) statistical method.

### Statistical analysis

All experiments previously described were performed using three independent cultures, and each condition was evaluated in triplicate. The data normality for all assays was evaluated using the Shapiro-Wilk test (*p>0*.*05*). Analysis of viability and infected cell percentage was done using the t-student test (*p<0*.*05*), while protein and AChE concentration data were analyzed using the Mann-Whitney test (*p<0*.*05*). Analysis of plaque assays data was performed using the non-parametric analysis of variance (ANOVA) known as Kruskal–Wallis test, followed by the Mann–Whitney U test (*p<0*.*05*). For the TEER and permeability assays, ANOVA and Kruskal-Wallis analysis were performed, followed by the post hoc tests Tukey and Mann–Whitney (p<0.05). Also, we performed a Spearman rank correlation test for the comparable group of data for TEER results (NEC vs IC; *P (-) vs P (+); NEC vs EVs (+)*_*UV*_ vs *EVs (+)*_*4G2*_ *vs EVs(-)*; and *P (+)* vs *IC*), and we also correlate the TEER and DB results for each group. Finally, for the CBA assay, the non-parametric *t*-test known as Wilcoxon signed Rank test was used to determined statistical differences among the evaluated data (p<0.05).

## Results

### Infected U937 cells produced vesicles heterogeneous in composition and density

**Fig 2:**
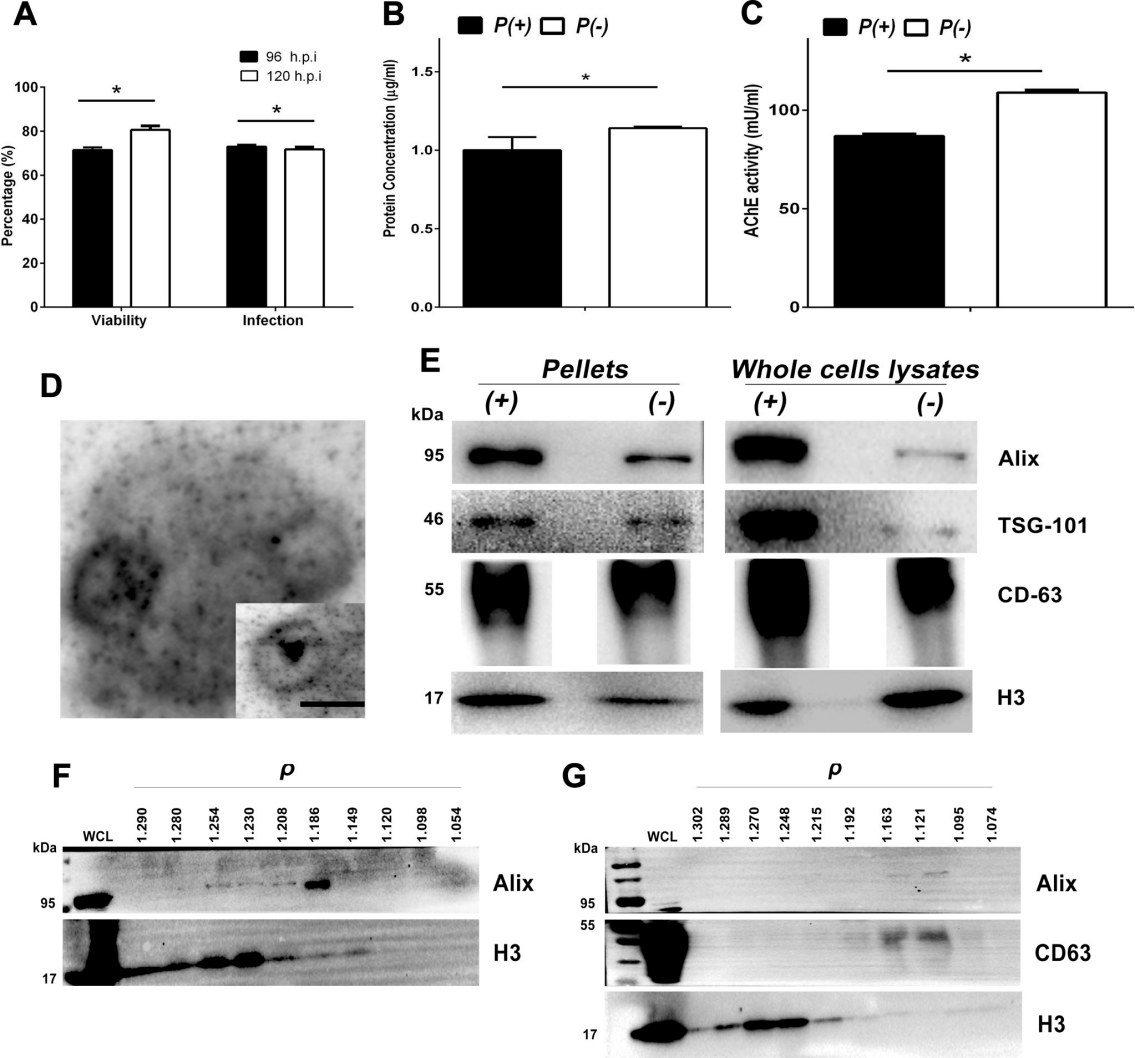
Infection percentages, cell survival, production, and characterization of the *P (+)* and *P (-)* pellets. Infected U937 cells were evaluated by IF to determine the infection percentage and survival with Calcein AM at 96 and 120 h p.i. For this, ten replicates were evaluated by each condition of three independent experiments (A). Supernatants were collected at 120 h p.i. and then ultrafiltrated and ultracentrifuged. Protein concentration was determined by BCA technique (B) and the AChE activity was evaluated (C). Significant differences were found between conditions according to a using a Mann-Whitney test (*p<0*.*05*). The morphology of the EVs of infected cells corresponded to classical cup-shape double-membrane vesicles. Bar: 100 nm. (D). 15 μg/μL of *P (+)* and *P (-)* or 10 μg/μL of whole cells lysates from infected (*WCL (+))* and uninfected cells *(WCL (-)*) were processed by SDS-PAGE and characterized by WB for detecting proteins Alix (95 kDa), TSG-101 (46 kDa), CD63 (55–25 kDa) and H3 (17 kDa) (E). Alix and TSG-101 were observed with higher intensity in the pellets and lysates of infected cells. A representative WB of 3 experiments per condition is shown. Finally, a sucrose gradient was used to establish the density of EVs from *P (+)* (F) and from *P (-)* (G). The linear gradient from sucrose 2.5 to 0.5 M was loaded with *P (+)* or *P (-)* and ultracentrifuged, then each fraction was collected, pelleted and processed by WB. In this way it was observed that both the *P (+)* and the *P (-)* presented heterogeneous vesicles with densities (ρ) between 1.14 to 1.2 g / Ml that were positive for Alix which corresponded to exosomes and between 1.24 to 1.28 g / mL positive for H3 that corresponded to apoptotic bodies. Whole cell lysates *(WCL)* from infected or uninfected cells were used as positive control. A representative WB is shown.

The cell line U937 had percentages of infection with DENV of 73% and 71.7% at 96 and 120 h p.i., respectively, with cell viabilities of 72% and 80% and statistically significant differences (*p<0*.*05*) at the evaluated times (Fig 2A). We found significant differences in protein concentration and AChE activity (*p<0*.*05)* between pellets collected from infected and mock infected cells, suggesting that the infection modified the secretion of vesicles in these cells (Fig 2B and 2C).

By electron microscopy it was observed that the vesicles obtained in the pellet of the infected cells (*P (+))* showed some round structures with an approximate diameter of 100 nm and a central depression that confers the characteristic cup shape [2] (Fig 2D).

When evaluated with WB, tetraspanin CD63 and the proteins of the ESCRT complex Alix and TSG-101 were detected in both *P (+)* and *P (-)*, suggesting the endosomal origin in most of these. Interestingly, complex ESCRT proteins were more evident in the sediments and lysates of infected U937 cells compared to that observed in uninfected cells, suggesting that DENV infection induced overexpression of these proteins and their packaging in the vesicles released (Fig 2E).

The mass spectrometry analysis of *P (+)* demonstrated the presence of common proteins, such as enolase, ubiquitin, tetraspanins, HSC70, annexins and heparan sulfate proteoglycans. Proteins with a role in the immune response were also detected, such as IL-6, C3 complement protein, metalloproteinase inhibitor, IgG1 heavy chain and galectin-3 sequester receptor binding protein (Table 1).

**Table 1:** Nano LC-MS / MS analysis identified in the EVs of P (+) and P (-) from macrophages U937.

**Pellets from infected U937 cells *(P(+))***			
**PROTEIN**	**PEPTIDES**	**MATCH**	**MAX SCORE**
SPARC	9	16	95,2
Alpha-1 Antitrypsin	5	11	65,1
Cathepsin L1	5	13	77,8
Ubiquitin/actin fusion protein	5	10	52
Actin	4	4	58,3
Poly-Ubiquitin	3	10	52
Heat Shock Cognate 70	3	5	58,7
14-3-3 gamma	3	6	70,6
Enolase	3	4	77,8
14-3-3 theta	3	4	99,5
Niemann-Pick Disease, Type C2	3	5	60,9
Metalloproteinase Inhibidor	2	4	89,7
Fibronectin	2	4	66,8
Dipeptidyl-Peptidase 7	2	1	71
PDI	2	2	79,7
Alix	2	2	85,5
Cysteine protease/legumain	1	1	65,5
IL6	1	1	65,1
CD81	1	2	77,4
Neuroserpin/serpin1	1	2	63,8
Clusterin	1	2	62,6
Calsyntenin	1	1	66,6
Neurosecretory protein vfg prec	1	1	67,2
Cathepsyn z	1	2	54,1
C3	1	1	60,9
Annexin A2	1	1	56,2
Follistatin-Related Protein 1 Precursor	1	1	68,1
Immunoglobulin Heavy Constant gamma 1	1	4	75,1
Aldo-ketoreductase	1	1	74
Anexin A1	1	1	50
Alpha-N-Acetylglusaminidase	1	2	70,2
Glyceraldehyde-3-Phosphate Dehydro	1	1	52,8
**Pellets from uninfected U937 cells *(P(-))***			
**PROTEIN**	**PEPTIDES**	**MATCH**	**MAX SCORE**
Sparc	6	11	95,2
Cathepsin L1	5	10	77,8
Alpha-1 Antitrypsin	4	4	63
Polyubiquitin	2	6	52
Cysteine protease/legumanin	2	4	63,4
Enolase	1	3	70,2
Alix	1	1	63
Annexin A5	1	1	56,6
Transthyretin	1	1	63,8
Serpin1	1	5	60,4
Heparan sulfate	1	1	55,4
Cathepsin Z	1	2	54,1
Calsyntenin 1, Isoform CRA	1	1	66

Proteins and number of peptides identified by nano LC-MS / MS analysis of the EVs of uninfected *(P (-))* and infected *(P (+))* macrophages.

Vesicle density flotation on a sucrose gradient after UC allowed the separation of different membranous structures. The fractions at different densities were recovered and analyzed again via WB. In the pellet of the infected cells *P (+)*, the 1.12, 1.19 and 1.2 g/mL fractions showed vesicles positive for Alix and weakly for H3 suggesting the presence of exosomes. In the fractions 1.23, 1.25 and 1.28 g/mL, the vesicles were highly positive for H3 and for Alix with a lighter band (Fig 2F), which suggests the presence of ABs.

The pellet of the uninfected cells (*P (-))* showed a similar protein profile where Alix and CD63 exosome markers were detected mainly at densities of 1.16, 1.19 and 1.21 g/mL, while at densities 1.24 and 1.28 g/mL, more H3 was detected (Fig 2G), revealing that infected and uninfected U-937 line macrophages secrete different vesicle populations, corresponding to low density exosomes containing Alix (1.14 to 1.25 g/mL) and high density ABs containing H3.

### EVs from infected U937 macrophages transport NS3 protein, but not DENV

**Fig 3:**
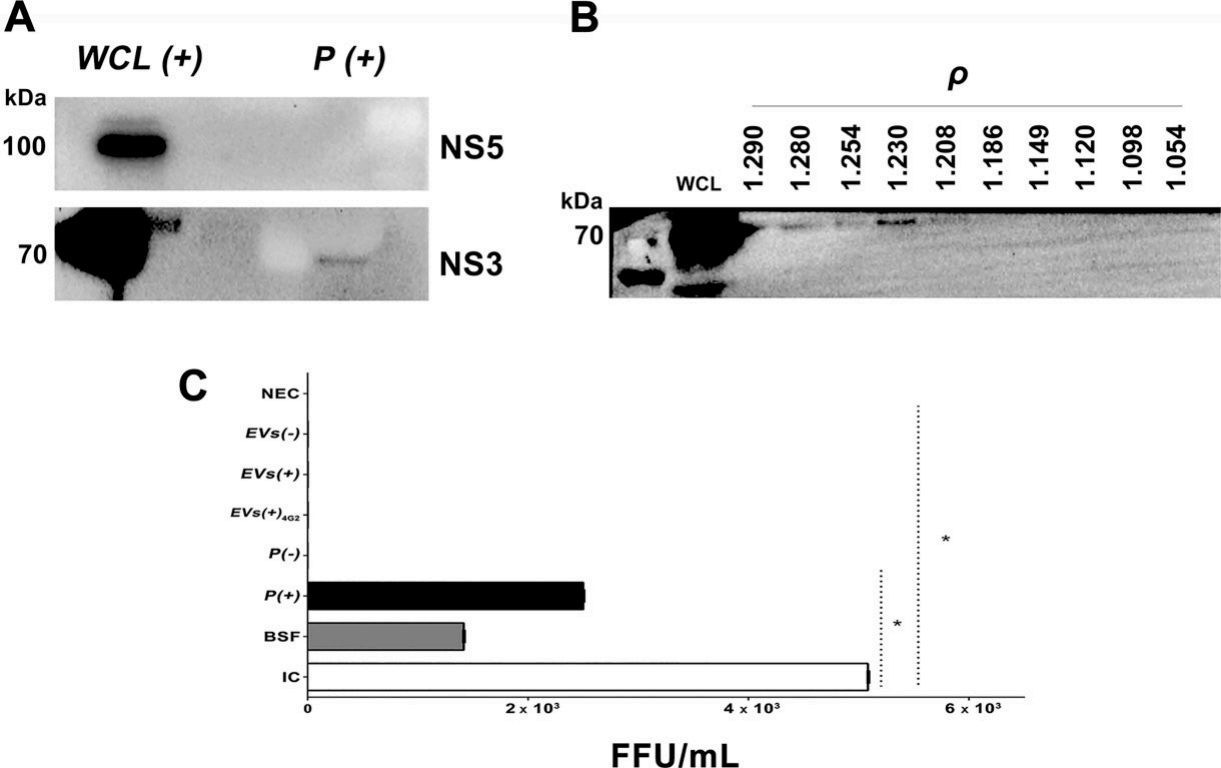
EVs of infected macrophages contain the NS3 protein of DENV-2 but not infectious material. (A). 10 μg/μL of an extract of infected cells (*WCL (+))* or 15 μg/μL of *P(+)* were processed by SDS-PAGE and WB to detect the DENV NS5 (105 kDa) or NS3 (70 kDa) proteins. These samples were positive only for NS3 protein. WB representative of two tests per condition is shown. (B). The EVs of infected cells with densities (ρ) between 1.23 to 1.28 g / mL were positive for the NS3 protein, corresponding to ABs (H3 positive). As control we used *WCL (+)*. (C). After UC, the EVs of the uninfected and infected macrophages were processed by immunoprecipitation (IP) and the resulted vesicles from uninfected (*EV (-)*) or infected (*EV (+)*) pellets were inoculated on LLC-MK2 cells and then foci formation were evaluated by immunoperoxidase at 72 h p.e. As controls the following conditions were evaluated: non-exposed cells (NEC); LLC-MK2 cells infected with DENV-2 MOI: 0.2 (IC); cells inoculated with beads free supernatants *(BFS)* obtained after the IP process; cells inoculated with the *P (-)* or *P (+);* vesicles of U937 infected cells, purified by IP and treated with 4G2 antibody (*EVs (+)*_4G2_) or vesicles of U937 non-infected cells (*EVs(-)*) purified by IP. Viral antigen was detected when LLC-MK2 cells were exposed to DENV-2, *BFS*, and *P (+)*. Mean of focus-forming units of two independent experiments by duplicate is shown. Data was analyzed using the Kruskal–Wallis test, followed by a Mann–Whitney test, with p<0.05.

Some reports have shown that the infected cells release vesicles that contain proteins or genetic material of the virus; however, it is not known if this happens during the infection with DENV. The pellets of the infected cells (*P (+))* were evaluated by WB and the NS3 protein was detected, but not NS5 (Fig 3A). The NS3-positive vesicles had densities of 1.23 and 1.28 g/mL that were also positive for ABs H3 marker (Fig 3B). These findings are novel, and we do not know their function, however, we suggest that this protein in these vesicles may be involved in the modulation of the cellular response during infection.

We also evaluated the infectious capacity of the vesicles purified from the U937 macrophages supernatants *(EVs (+))*. For this, we purified the vesicles by IP using magnetic beads coated with anti-CD63. The recovered vesicles were positive for NS3, Alix and H3, although the latter was faintly detected (S1 Fig). Interestingly, these vesicles, when inoculated on LLC-MK2 cells, were not infectious according to the classic focus forming units assay (FFU), whereas the unpurified pellet and the BFS inoculated onto LLC-MK2 cells yield countable foci and reach titers of 2.5 × 10^3^ FFU/mL and 1.4 × 10^3^ FFU/mL, respectively (Fig 3C).

These results indicate that the *P (+)*, are heterogeneous vesicles constituted by exosomes, ABs and -maybe- other small vesicles (called hereafter EVs) and free and infectious viruses. In consequence, it is plausible to say that the IP process allowed obtaining EVs enriched of exosomes positive for the Alix protein. Also, the observed results indicate that the NS3 protein is present mainly in the ABs, but these structures do not contain infectious material.

### EVs from infected macrophages affected the endothelial cells EA.hy926

To evaluate the effect of the previously characterized EVs, they were inoculated on the endothelial cell line EA.hy926 seeded on transwell® inserts. The experiments were initiated when the EAhy.926 cell monolayer reached a TEER value of 25 Ω/cm^2^ (Time 0). In general, all the evaluated conditions induced an increase in TEER during the evaluated times.

**Fig 4:**
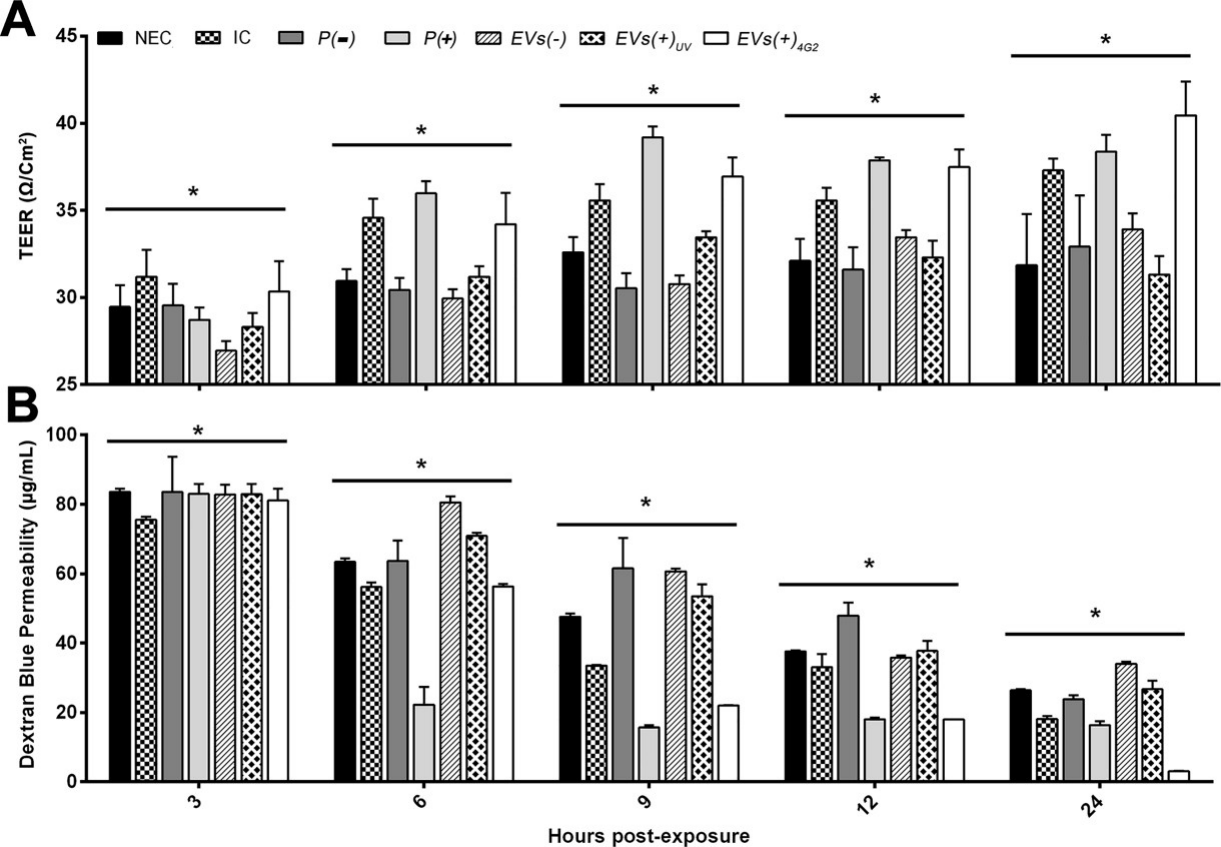
Changes in TEER and permeability of EA.hy926 cells exposed to EVs or pellets from infected or uninfected macrophages. (A). The EA.hy926 cells seeded on transwell® inserts showed a TEER of 25 Ω/Cm^2^ at time 0. The controls: non-exposed cells (NEC), cells exposed to IP vesicles from uninfected U937 macrophages *(EV (-))* or EVs from U937 infected macrophages and irradiated UV (*EVs (+)*_*UV*_*)*, presented an increase in the TEER between 2 and 9 Ω/cm^2^ in the course of the experiments, while the EA.hy926 cells infected with DENV-2 (MOI 0.2) (IC) reach 12 Ω/cm^2^ at 24 h p.e. Interestingly, TEER increased significantly (up to 15 Ω/cm^2^) when EA.hy926 cells were exposed to EVs obtained from infected U937 macrophages (*EV (+)*_*4G2*_ or *P(+))* (B). In parallel, the concentration of dextran blue 2000 (DB) in the lower chamber of the inserts was evaluated. At time 0, the DB concentration was 27 μg/ml, then at 3 h it increased to 80 μg/ml and at 6 h p.e some conditions decreased the permeability up to 60 μg/ml; in particular endothelial cells exposed to *P(+)* had a significant decrease of DB concentration (25 μg/ml) at this time. From 9 to 24 h p.e, all conditions started to show an important diminish of DB concentration specially when cells were exposed to *EVs (+)*_*UV*_ or *P (+)* compared to *EVs (-)* or *P (-)* and the other controls. The mean and standard deviation of 3 to 5 transwell® inserts are shown per each condition. Data that according to the Shapiro-Wilk normality test were considered normal were analyzed using an ANOVA analysis and a Tuckey post-hoc test. Otherwise, non-parametric ANOVA test known as Kruskall-Wallis was performed followed by the Mann-Whitney U test. All statistical significance were obtained using a p value <0.05. Significant differences in the conditions evaluated in each of the evaluated periods are shown.

When the EC cells were non-exposed (NEC), or exposed to the control EVs of infected macrophages irradiated with UV light (*EVs (+)*_*UV*_), TEER values showed a similar increase that varied between 3–9 Ω/cm^2^ from 3 h to 24 h post exposure (h p.e.) that were statistically significant (*p <0*.*05*). Similarly, cells exposed to the controls pellet of uninfected macrophages U937 (*P (-)*) and EVs from uninfected U937 cells purified by IP *(EVs (-))* showed a rise in TEER equivalent to 6 Ω/cm^2^ that was stable from 6 to 9 h p.e. and had a subsequently slight increase from 12 to 24 h p.e (*p <0*.*05*). Moreover, cells exposed to DENV-2 (IC) showed a time-dependent boost in TEER values that range between 6–12 Ω/cm^2^ during the evaluated time lapse (*p <0*.*05*). The latter may indicate that the inoculated virus by itself does not cause an endothelial damage at least in the evaluated times and MOI (Fig 4A).

On the other hand, cells exposed to pellets of infected macrophages (*P (+)*) and EVs of infected macrophages treated with neutralizing antibody 4G2 (*EVs (+)*_*4G2*_), presented an important increase in TEER values (*p <0*.*05*). EC exposed to *P (+)* had a rise in TEER that varied between 7–15 Ω/cm^2^ from 3 to 9 h p.e, remaining relatively stable for the rest of the evaluated times; meanwhile, *EVs (+)*_*4G2*_ had a time-dependent increase in TEER values that range between 3–5 Ω/cm^2^ in each evaluated time, reaching the highest TEER value at 24 h p.e. equivalent to 40 Ω/cm^2^ (*p <0*.*05*) (Fig 4A).

On the other hand, a Spearman correlation test for TEER data was performed. In general, these results showed positive correlations for all evaluated variables, with moderate (*r = 0*.*4–0*.*69*), strong (*r = 0*.*7–0*.*89*) or very strong correlation (*r = 0*.*9–1*), according to the interpretation ranks published by Schober et al, 2018 [47]. (S1 Table).

Importantly, the correlation results for *NEC* vs *EVs (+)*_*uv*,_ showed a very strong positive correlation (*r = 1*), showing that particles from U937 infected macrophages treated with UV light cause the same TEER changes than non-exposed cells, proving that these controls are comparable. When evaluating the *IC vs NEC*, *P (-)* vs P *(+)* or *EVs (+)*_*uv*,_ vs EVs (+)_4G2_, it was clear that the virus or the vesicles released from U937 infected macrophages (immunoprecipitated or not) caused a different impact on TEER results than their respective controls. Also, it seemed that treating the vesicles from infected macrophages with UV light caused a different impact on TEER than the vesicles obtained directly from non-infected U937 cells (*EVs (-)*), S2 Fig.

On the other hand, the correlation results showed that regardless of whether endothelial cells are exposed to complete viral particles (IC) or vesicles from infected U937 macrophages (*P (+)* or *EVs (+)*_*4G2*_), the effect observed in TEER is very similar, indicating that it is not necessary for the virus to have contact with the endothelial cells to cause alterations in these, being the information carried by the vesicles from cells that did have direct interaction with the virus enough for this, even if the vesicles were treated with the 4G2 antibody. Additionally, it was observed that the immunoprecipitation process implemented to enriched the exosomes population of the obtained vesicle set, did not induce different changes on the endothelial cells than the non-precipitated vesicles (*EVs (-)* vs *P(-)*, *r = 1)*, S2 Fig.

Under the same experimental conditions, the permeability of the model was evaluated. At time 0 the mean concentration of DB in the lower chamber was 27 μg/mL, corresponding to the basal system permeability. At 3 h p.e., a permeability increase of the system was recorded with an average of 80 μg/ml DB in the lower chamber, possibly associated with the change of medium and the manipulation of the system. From this time on, all permeability values started to diminish, e.g. at 6 h p.e, the NEC and the infected cells (IC) showed a decrease in the system permeability registering DB values of 62 and 59 μg/mL respectively. Nevertheless, the cells exposed to *EVs (-)*, or *EVs (+)*_*UV*,_ maintained a high permeability (DB, 80 and 75 μg/mL respectively). Importantly, during the same evaluated time *P (+)* and then *EVs (+)*_*4G2*_ (at 9 h p.e) had an outstanding decrease in DB values showing a permeability equivalent to 20 μg/mL or less, that remained relatively stable during the other evaluated times (Fig 4B). Overall, at 9, 12 and 24 h p.e., the system permeability was gradually reduced in all the experimental conditions evaluated, including the infected cells, until reaching at 24 h.p.e., similar or lower values than those obtained at time 0 (Fig 4B). These differences were statistically significant among them and between groups according to the Mann Whitney test with values of *p <0*.*05* (Fig 4B).

Also, a Spearman correlation test was performed, comparing the results obtained for TEER and AD for each evaluated condition (S1 Table). The latter confirmed for all conditions that if TEER values increase, the system permeability tends to decrease, just as expected (S3 Fig). Together, these results indicate that the EVs obtained from infected U937 macrophages modulate the endothelial response.

### The EVs produced by U937 cells induce endothelial activation, which increases with DENV presence

**Fig 5:**
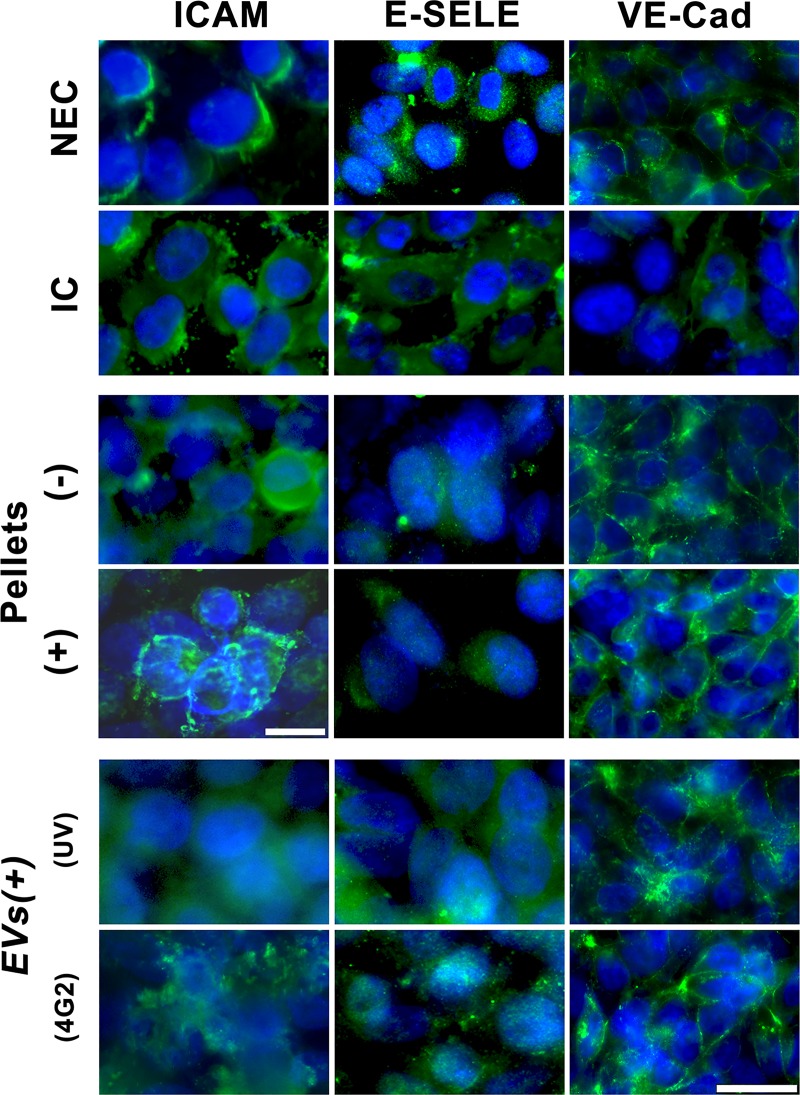
EVs obtained from U937 cells increased the expression of adhesion molecules in endothelial cells EA.hy926. EA.hy926 cells seeded on transwell® inserts were exposed to the different evaluated conditions for 24 h p.e., then the cells were processed by immunofluorescence (IF) to detect the proteins VE-Cad, E-Selectin (E-Sele) or ICAM. Cells infected with DENV-2 at MOI 0.2 (IC) showed a slight decrease in the expression of VE-Cad, while in cells exposed to *P (+ or -)* or *EV (+ 4G2 or UV)* showed a bright fluorescence pattern that delimits the cells. E-Selectin (E-sele) was detected in the cytoplasm of IC and slightly in endothelial cells exposed to *EVs (+) 4G2* or *UV*. Similarly, in the infected cells ICAM was detected in the cytoplasm, while in cells exposed to *P (+)* or *EVs (+) 4G2* the labeling was observed on the cell surface. Photomicrographs are representative of two duplicate experiments. Bar: 10 and 50 μm.

Interestingly, it was observed that these modifications in resistance and permeability were not associated with changes in the expression and location of adherent binding proteins such as VE-cadherin (VE-Cad). In this regard, when the endothelial cells were exposed to the virus, an intensity decrease of VE-Cad was observed, while the cells exposed to the pellets (*P+*) or EVs of infected U937 macrophages (*EV(+)*_*uv*_ *or EV(+)*_*4G2*_*)*), showed an increase of the fluorescence signal for VE-Cad delimiting each of the cells. The same situation was observed when the cells were exposed to irradiated EVs with UV light (*EVs (+)*_*UV*_*)*, and in the other controls (Fig 5).

The molecules E-selectin (E-sele), ICAM and VCAM were also evaluated in EA.hy926 cells after exposure to EVs. E-sele was detected in the cytoplasm with a low or medium fluorescence in cells infected with DENV-2 (IC), as in cells exposed to *EVs (+)*_*4G2*_ and *EVs (+)*_*UV*_. ICAM slightly increased the fluorescence only in IC cultures, whereas VCAM (protein or mRNA) was not detected under these experimental conditions (Fig 5). By qRT-PCR 15-fold upregulation was observed for ICAM-1 in infected endothelial cells, and a 6-fold increase was observed in cells exposed to *P (+)* compared to those exposed to *P (-)*.

On the other hand, cytokines levels were determined. Initially, input levels of the immune mediators carried from the infected U937 cell line to the endothelial cells were evaluated. Input levels of TNF-α, IL-10, IFN- α, IL12p70 and IFN-γ were always below the limit of detection. Regularly, input levels of IP-10, MCP-1, RANTES, IL-8 and IL-6 were 109, 1726, 1406, 606, and 6 pg/mL respectively. The latter data were used to normalize the results obtained from the supernatants of endothelial cells exposed to *P (+)* or *EVs (+)*.

**Fig 6:**
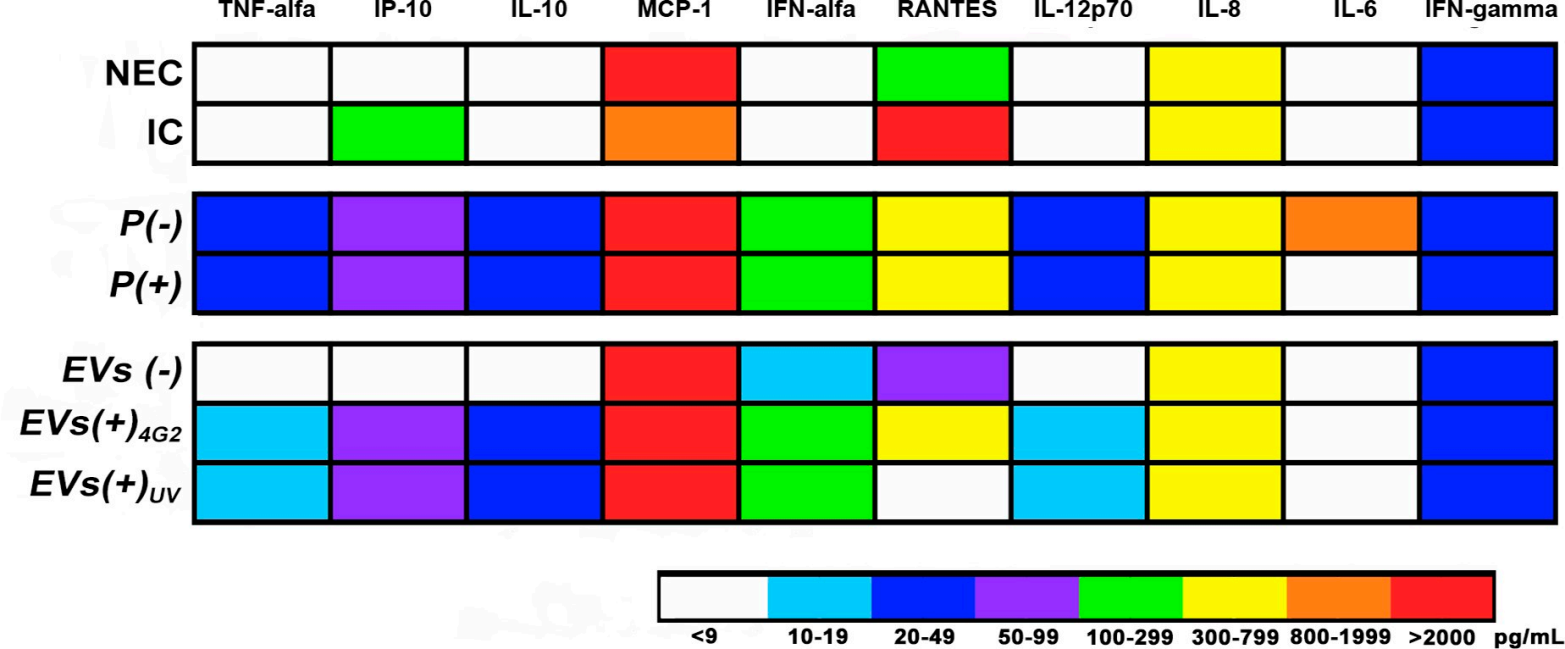
Quantification of soluble mediators produced by endothelial cells EA.hy926 exposed to pellets (*P+* or *P-*) or EVs. Heat map showing the variations in the concentration (pg/mL) of cytokines and chemokines produced by EA.hy926 exposed to the pellets or EVs of infected or uninfected macrophages. The average concentration of two independent cultures is shown in duplicate. The evaluated conditions were: Non-exposed cells (NEC); infected endothelial cells with DENV-2 MOI: 0.2 (IC); pellets from non-infected macrophages *(P(-))*; or from infected macrophages *(P(+))*, EVs purified from non-infected macrophages *(EVs (-))*; or from infected macrophages *(EVs (+))* treated with 4G2 antibody *(EVs (+)*_*4G2*_*)* or UV irradiated (*EVs (+)*_*UV*_).

The measurement of immune mediators in endothelial cell supernatants showed that compared to the NEC control, DENV-2 infection induced an increase in IP-10 (109 pg/mL), IL-6 (6 pg/mL), and a statistically significant increment in RANTES (2640 pg/mL, p<0.05) as well as a slight reduction in IL-8 levels (688 pg/mL in NEC vs 605 pg/mL in IC) and a significant reduction in MCP-1 levels (1726 pg/mL in IC vs the NEC control that presented 5727 pg/mL, p<0.05). Other soluble mediators were below the limit of detection (2.4 pg/mL). Although, the levels of IFN-α (284 pg/mL, IL-10 (41 pg/mL), IP-10 (80 pg/mL) and RANTES (564 pg/mL) were similar for the endothelial cells exposed to *P(+)* and *P(-)*, the exposure to *P (+)* induced the secretion of TNF-α (42 pg/mL), IL-6 (920 pg/mL) and a statistically significant increase for IL-8 (750 pg/mL, p<0.05), when compared to the cells exposed to *P (-)* (Fig 6).

Interestingly, exposure to *EVs (+)*_*UV*_ induced higher levels of TNF-α, IL-12p70 and IL-8 (12, 17 and 747 pg/mL, respectively) and a statistically significant increase (*p<0*.*05*) of IP-10 (68 pg/mL), IL-10 (33 pg/mL) and IFN-α (244, pg/mL) compared to cultures exposed to *EVs (-)*. Conversely, RANTES levels were higher in cells exposed to *EVs (-)* than in cells treated with *EVs (+)*_*UV*_ (75 pg/mL vs 8 pg/mL, *p<0*.*05*). Moreover, treatment of EC with *EVs (+)*_*4G2*_ produced an increase in TNF-α (11 pg/mL) and IL-12p70 (10 pg/mL) as well as a significant increment in IP-10, IL-10, IFN-α, RANTES and IL-8 (68, 31, 242, 511 and 679 pg/mL, respectively, *p<0*.*05*). The latter changes did not occur when cells where treated with *EVs (-)*. In all cases, the concentrations of MCP-1 were higher than 11,000 pg/mL, regardless the type of EVs or if they were obtained from infected or uninfected cells. Finally, the levels of immune mediators were similar in the endothelial cells exposed to *EVs (+)*_*4G2*_ or *EVs (+)*_*UV*_, with the exception of RANTES (higher in cells exposed to *EVs (+)*_*4G2*,_ *p<0*.*05*) and IL-8 (higher in cells treated with *EVs (+)*_*UV*_) (Fig 6).

These results demonstrate that the purified EVs induce the activation and secretion of some inflammatory mediators by endothelial cells EA.hy926, and this response is slightly increased even when the virus is present in low concentrations. Interestingly (with some exceptions), cytokines and chemokines secreted by endothelial cells were similar after exposure to *EVs (+)*_*4G2*_ or *EVs (+)*_*UV*_, and these levels were significantly higher than in non-exposed control cells or exposed to *EVs (-)* (Fig 6), suggesting that the EVs of infected U937 macrophages -in the absence of the virus-, modulate the endothelial response.

### EVs secreted by U937 macrophages transport different miRs

**Fig 7:**
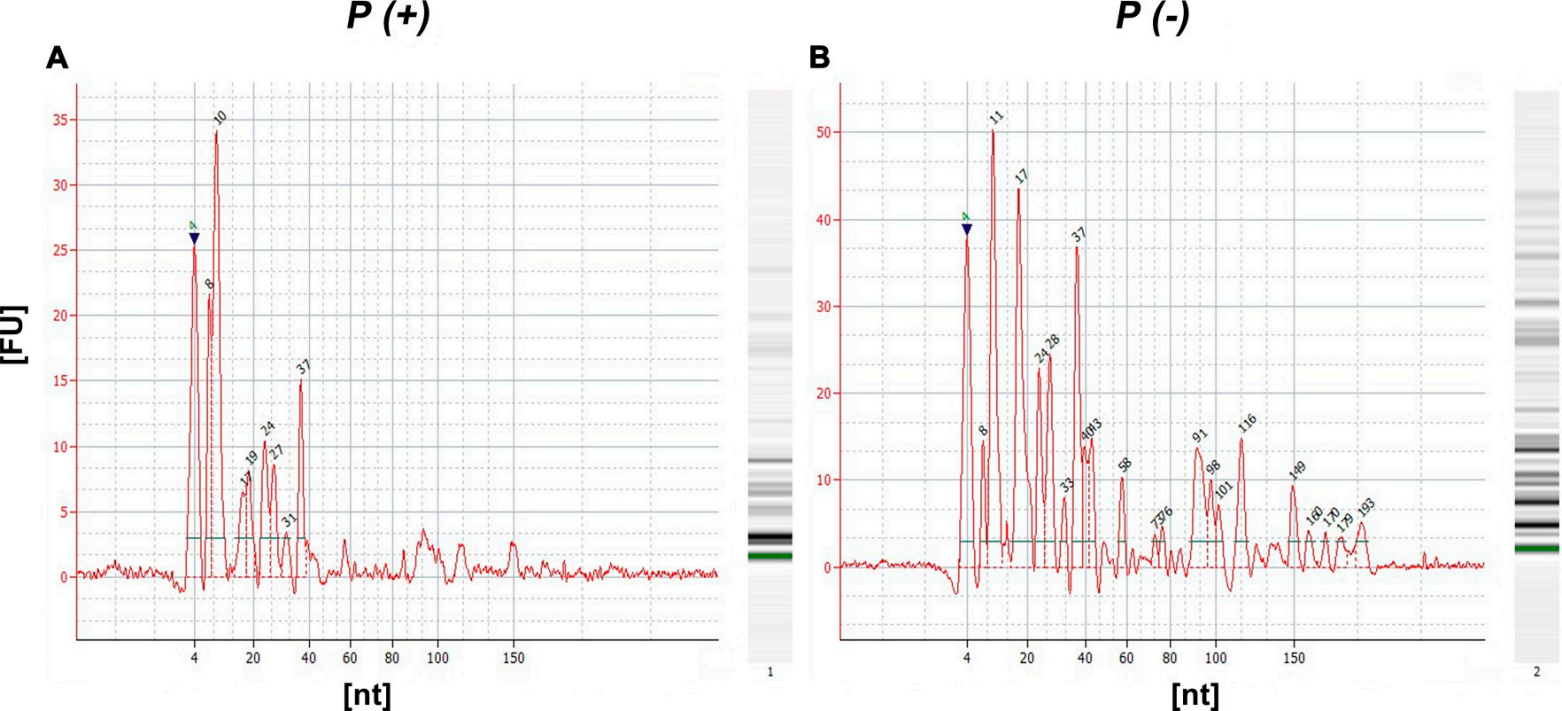
Exosome enriched EVs obtained from DENV infected and non-infected U937 transport different small RNA. Pellets obtained from the infected P(+) (A) and uninfected P(-) (B) macrophages were processed for the extraction of small RNA, allowing the identification of different kinds of molecules such as 15 to 25 nt length micro RNA (miR), transfer or tRNA fragments (between 30 and 79 nt) and rRNA fragments (larger than 80 nt). The amount of each RNA species measured per fluorescence units (FU) was higher in P (-).

The RNA profile of different identified sRNAs in both *P (+)* and *P (-)* conditions was obtained (Fig 7). In Figures Fig 7A and 7B is shown the identified profile for miRs (15–25 nt), tRNAs (26–80 nt) and rRNAs (> 80 nt).

The miRNA sequencing resulted in the identification of a total of 11 miRNAs (Table 2). Four miRNAs (miR-27b-3p, miR-3135b, miR-30a-5p, miR-21-3p) were expressed just in *P (-)*, three miR-181a-5p, miR-4301 and miR-4652-3p) in *P (+)* and four (miR-22-3p, miR-21-5p, miR-92a-3p and miR-191-5p) were detected in In both *P (+)* and *P (-)*,however these four were, interestingly, more abundant in *P (-)* (Table 2).

**Table 2:** Common or specific miRs identified in the EVs of *P (-)* and infected *P (+)* macrophages U937 with DENV-2.

*P(-)*		Both	Counting		*Fold change*	*P(+)*	
Counting			*P(-)*	*P(+)*		Counting	
**hsa-miR-27b-3p**	173.189	**hsa-miR-22-3p**	632.751	200.006	-0,50	**hsa-miR-4301**	115.676
**hsa-miR-3135b**	122.420	**hsa-miR-21-5p**	528.838	119.702	-0,65	**hsa-miR-4652-3p**	85.670
**hsa-miR-30a-5p**	101.132	**hsa-miR-92a-3p**	103.647	47.766	-0,34	**hsa-miR-181a-5p**	53.179
**hsa-miR-21-3p**	67.140	**hsa-miR-191-5p**	101.621	55.592	-0,26		

Table 2 presents the miRs that had the highest number of counting after the sequencing analysis. The table lists the common miRs, and in an independent manner shows the miRs identified specifically in the EVs obtained from uninfected macrophages U937 *(P (-))* or infected macrophages *(P (+))*.

**Fig 8:**
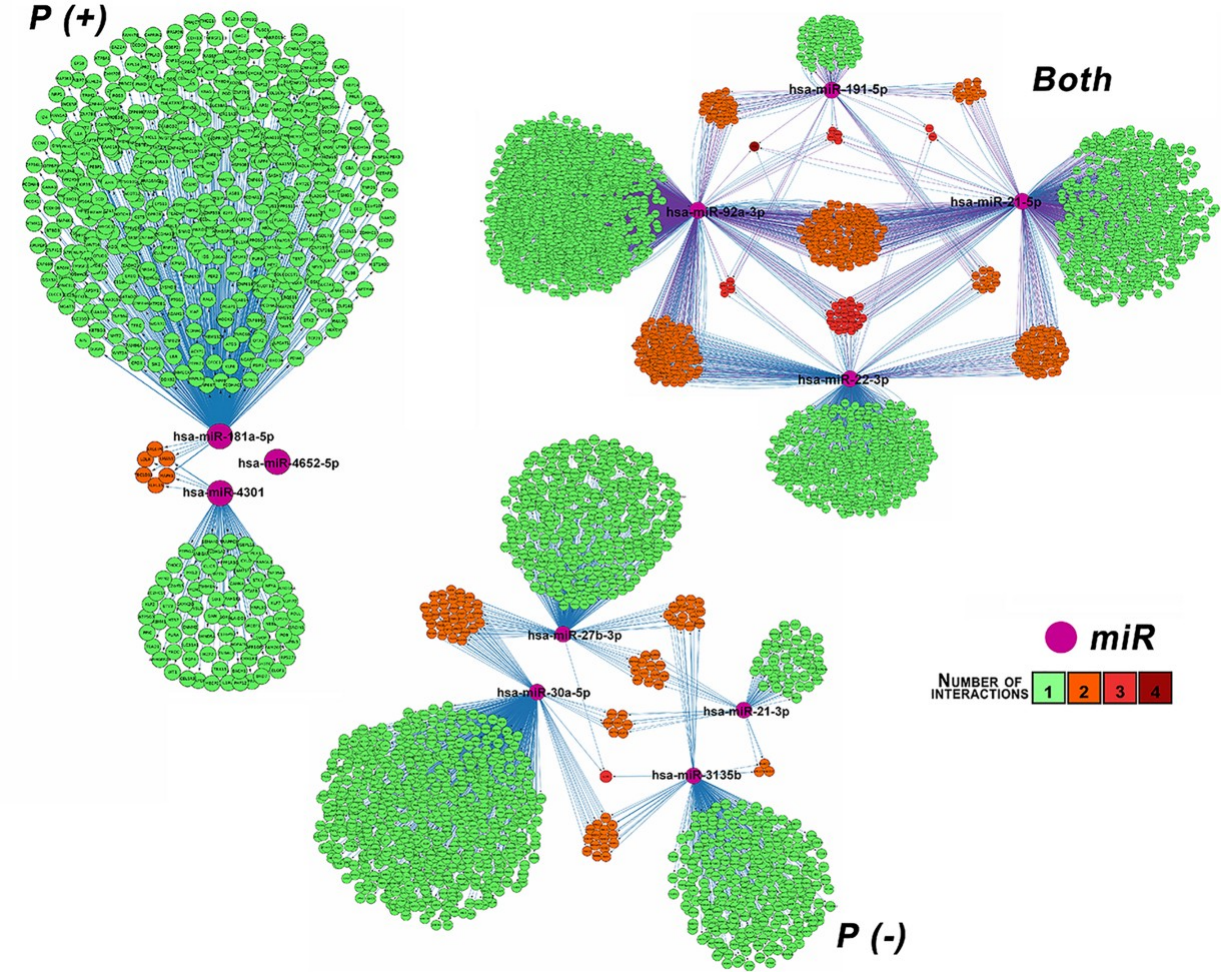
Regulatory network of miR-targets. The miR-gene regulation networks were built by miRs expressed in vesicles of infected U937 cells (*P (+)*), non-infected U937 cells (*P (-))* or in both conditions. miRs are represented in violet and target genes are displayed in bright green, light orange, dark orange and red circles depending on the number of regulatory interactions between the miR and its target or targets. Bright green indicates one regulatory relationship, light orange, dark orange and red indicates two, three and four regulatory relationships, respectively.

The target genes for each expressed miR in *P (+)* (601 targets) (S2 Table), *P (-)*(1365 targets) (S3 Table) and in both conditions (3344 targets) (S4 Table) were predicted and their regulatory networks were constructed using Cytoscape (Fig 8). For one of the three expressed miRs (hsa-miR-4652-5p) in *P (+)* condition, it was not possible to predict any target.

In Fig 8, miRs are represented by violet color. Given that certain targets genes are regulated by more than one miR, targets colors are based on the number of regulatory relationships, thus bright green indicates one regulatory relationship and light orange, dark orange and red color indicates two, three and four regulatory relationships respectively, for example, PURB gene was regulated by hsa-miR-22-3p,hs a-miR-21-5p, hsa-miR-191-5p, and hsa-miR-92a-3p and is represented by the red color.

GO enrichment analysis was conducted and it resulted in 16 significantly enriched terms for *P (+)* condition (S5 Table), 49 for *P (-)* condition (S6 Table) and 68 for both conditions (S7 Table). The *P (+)* enriched terms are mainly related to processes such as cell adhesion, cell cycle DNA integrity checkpoint and regulation of cell communication S5 Table). On the other hand, the *P (-)* enriched are related to regulation of cell cycle, more specifically in regulation of protein kinase activity and cyclin-dependent protein kinase. Other processes include transport, regulation, modification and efflux of lipids, organization and transport of pigment granules (S6 Table). Additionally, miRs expressed in both conditions were mainly involved in the metabolism and catabolism of lipids, small molecules and organic acids and energy coupled processes (S7 Table).

## Discussion

The proposed experiments allowed the evaluation of the EVs secreted by U937 macrophages, a cell line highly susceptible to DENV infection and frequently used to study the macrophages role on infection and pathogenesis [31]. U937 produced EVs with a similar buoyant density to those previously described to exosomes and small apoptotic bodies [5, 48]. These structures contain Alix, TSG-101, CD63, H3, annexin, enolase and ubiquitin such as those purified using other cell types [49], but do not contain infectious material. For the first time, we reported that EVs with 1.20–1.28 g/mL density are positive to Alix and H3 and transport the viral NS3 protein. Interestingly, mock and infected U937 cells produced different EVs quantities (protein and AChE activity measurement).

The role that may have the presence of a DENV protein in these EVs is still not clear, although it has been reported that tetraspanin 189 bearing vesicles transport DENV in cytoplasmic prolongations of C6/36 cells [50] and other LC3-II positive large vesicles (2 to 5 μm) transport not only virus but RNA, and E, prM and NS1 proteins favoring the cell to cell transfer [51].

These results suggest that depending on the cell model, DENV infection can modulate cell death or defense processes such as apoptosis or autophagy mechanisms, kidnapping the cell transport of different types of vesicles, aimed to guarantee the movement and assembly of viral proteins and RNA and it’s intracellular or extracellular spreading. Other infection models like HCV [20] have shown that EVs participates in both inducing or evading the innate and acquired immunity. Despite there are no reports on free or vesicle associated DENV NS3 circulation during infection, it is possible that these NS3 positive vesicles could be involved in additional mechanisms of immune regulation, providing an alternative route for the antiviral response establishment.

For example, NS3 bearing vesicles could favor the MHC-I antigen presentation by immune cells after membranes fusion like it happens during HIV infection [52] or it could be presented through MHC-II because the macrophage origin of this vesicles, as it was demonstrated for a bacterial model [53]. In this case, the EVs are internalized by different mechanisms as endocytocis, macropinocytosis or phagocytosis and degraded in endo- or phagolysosome, facilitating the loading and presentation of peptides by immune cells [54].

This interpretation could explain those patients positive for NS3 specific immunoglobulins [55] and the broad CD8+ T lymphocytes populations recognizing NS3 epitopes [56, 57]. Findings reported here and others in different virus and cell models put the focus on the EVs and their proteins as a player in the host defense mechanisms or pathogenesis modulation. It is clear that these functions must be evaluated and confirmed; however, our findings, along with those previously reported, suggest the participation of EVs and non-structural viral proteins in the activation and defense of the host and their contribution to the disease pathogenesis.

Regarding the flavivirus NS3 immunomodulatory ability, it has been shown that HCV NS3 protein expressed after transfection inhibit the NF-κB signaling in host cells and promotes virus replication, interacting with LUBAC (linear ubiquitin chain assembly complex) and preventing the NEMO ubiquitylation [58]. Despite we did not confirm the NS3 download in endothelial cells, the changes in the immunofluorescence patterns of the adhesion molecules could be due to this protein or the miR transported by EVs.

On the other hand, the endothelial dysfunction during dengue disease -it is believed-, depends mainly on the inflammatory soluble mediators but also on the direct endothelial infection. Recently, it was demonstrated that EVs released by endothelial cells infected by DENV contain IFITM3 (interferon-inducible transmembrane protein 3) which induce an antiviral status in HeLa cells [59], suggesting a new endothelia activation/damage route where cytokines participate.

Interestingly, the cytokine and chemokines profile produced by polarized endothelial cells exposed to EVs induced ICAM expression and IL-6, TNF-α, RANTES and MCP secretion indicating a proinflammatory status possibly involved in the endothelial permeability alteration. Despite the low TNF-α, IL-6 and IL-8 levels and slight changes between conditions, this molecules at evaluated periods did not change the monolayer permeability, but induced an alertness status in endothelium, expressing adhesion molecules and chemokines (RANTES and MCP-1), similar to what happen after the endothelial exposition to vesicles produced by macrophages stimulated with IFN-α or LPS [60, 61].

Characterized EVs induced a slight endothelial activation, possibly determined also by the miR that they contain, and that we report for the first time in vesicles obtained from DENV infected cells. The miRs most frequently counted include the miR 92a, miR 21 and miR 191 are strongly associated to many biological processes involving endothelial cell processes such as angiogenesis [62, 63], tubular network formation [63], brain microvascular reparation [64], cell proliferation, migration and capillary formation [65]. Concerning to the miR 4301, it has a pro-apoptotic and antiproliferative role in tumors [66], however it has not been identified in DENV infections. The well-known miR 181, has a negative effect negatively in the proinflammatory response [67, 68] which is playing a role in endothelial activation during dengue disease. Likewise, analyzing the network effect of miR-4301, miR-4652 and miR-181a, it is presumable a composite impact in endothelial activation, since they as a whole, could regulate many genes involved in the barrier and structural functions of vessels [69, 70], a phenomenon that deserve more research. Interestingly, also was observed the modulation of other genes associated with lipid cycle and metabolism, these processes could play a key role and must be evaluated.

Our findings suggest that EVs (exosomes and AB) and other molecules from macrophages secretome such as HMGB-1 [67], S-100, IL-1α and IL-33 [68] could induce the endothelial activation process, introducing new players involved in cell response modulation during dengue infection. EVs are new key factors that can contribute to the understanding of dengue pathogenesis given the complex composition and pathways they use to change the cell response during DENV infection, as suggested by Hamlin et al., [71]. The hypothesis developed here propose that EVs secreted by DENV infected or activated cells, such as macrophages, are rapidly released and travel through the blood stream in an early manner, stimulating the endothelium and other immune cells, and contributing to the establishment of the first protective proinflammatory response during infection.

## Conclusion

We demonstrated for the first time that the DENV-2 infection of U937 macrophages induces a change in the production and composition of secreted EVs, which can transport the viral NS3 protein. Additionally, we demonstrated that virus-free EVs produced by infected cells induced early endothelial barrier changes and activation in a pro-inflammatory pattern, similar to the response induced by the virus itself. This response has a protective role during the early stages of infection, helping to maintain the endothelial monolayer integrity. A number of things remain to be clarified, such as i) whether other DENV-susceptible cells produce vesicles containing viral proteins, ii) the differences in EVs composition according to post infection periods or infecting serotype, iii) the effects of specific miRNAs transported by EVs from infected cells, and iv) the ability of NS3-positive EVs to activate antigen-presenting cells or T or B lymphocytes.

## Supporting information

S1 File Manuscript raw images. A pdf file that contains all the raw images of the blots presented in this paper. https://dataverse.harvard.edu/dataset.xhtml?persistentId=doi:10.7910/DVN/1Y17SG.
(PDF)

S1 Fig Reduction of apoptotic bodies (ABs) in pellets of infected macrophages U937 after immunoprecipitation (*EVs(+)*) with beads coupled to the CD63 antibody. Extracellular vesicles obtained from DENV infected and non-infected macrophages were pelleted by ultracentrifugation, processed by immunoprecipitation (IP) and then separated by electrophoresis. Purified vesicles from infected U937 cells *(EVs (+))* were positive to Alix and NS3 but slightly to H3. Control conditions were, infected whole cells lysate *(WCL (+))*, and the EVs bound to CD63 beads (EV + beads).
(TIF)

S2 Fig Graphic analysis for the Spearman correlation test for TEER results. Scatter plots with lineal regressions correlating the obtained TEER data in every evaluated condition. Graphs show for every comparable group of data (*NEC vs IC; P (-) vs P (+); NEC vs EVs (+)*_*UV*_ *vs EVs (+)*_*4G2*_ *vs EVs (-); and P (+) vs IC*) as well as the correlation coefficient (r) for them.
(TIF)

S3 Fig Graphic analysis for the Spearman correlation test comparing permeability and TEER results. Scatter plots with lineal regressions correlating the obtained results for TEER and permeability for all evaluated conditions showing the correlation coefficient (r) for them.
(TIF)

S1 Table Spearman correlation coefficient analysis for TEER and permeability. (XLSX)

S2 Table Regulatory network of miR-targets detected in vesicles of infected U937 cells (*P (+)*). (XLSX)

S3 Table Regulatory network of miR-targets detected in vesicles of uninfected U937 cells (*P (-)*). (XLSX)

S4 Table Regulatory network of miR-targets detected in vesicles of infected (*EV(+)*) and uninfected (*EV(-)*) U937 cells. (XLSX)

S5 Table GO Enriched terms for predicted genes targeted by the miRs detected in vesicles of infected U 937 cells (*P (+)*). To view the graphic map of Gene Ontology categories, go to the link located at the end of the file.
(XLSX)

S6 Table GO Enriched terms for predicted genes targeted by the miRs detected in vesicles of infected U 937 cells (*P (-)*). To view the graphic map of Gene Ontology categories, go to the link located at the end of the file.
(XLSX)

S7 Table GO Enriched terms for predicted genes targeted by miRs detected in vesicles of both conditions (infected (*EV(+)*) and uninfected (*EV(-)*) of U937 cells). To view the graphic map of Gene Ontology categories, go to the link located at the end of the file.
(XLSX)

## Abbreviations

AB: Apoptotic Bodies
AchE: Acetyl cholinesterase
BFS: Beads free supernatant
BSA: Bovine Serum albumin
C: capsid viral protein
DAB: 3'-Diaminobenzidine
DB: Dextran Blue
DENV: Dengue virus
E: Envelopment viral protein
EC: Endothelial cells
EBV: Epstein barr virus
Em: Emission
ESCRT: Endosomal sorting complexes required for transport
EVs: Extracellular Vesicles
EVs (-): EVs from uninfected U937 cells purified by IP
EVs (+): EVs from DENV-2 infected U937 cells purified by IP
EVs (+) _uv_: EVs from DENV-2 infected U937 cells purified by IP and treated with UV light
EVs (+) _4G2_: EVs from DENV-2 infected U937 cells purified by IP and treated with the 4G2 antibody
Ex: Excitation
FBS: Fetal Bovine Serum
g: Gravities
hCMV: Human Citomegalovirus
HCV: Hepatitis C virus
HIV: Human immunodeficiency virus
h. p.e: Hours post-exposure
h. p.i: Hours post-infection
IC: Infected cells
IF: Immunofluorescence
IP: imminoprecipitation
JEV: Japanese encephalitis virus
MOI: Multiplicity of infection
mrRs: micro RNAs
mRNA: messenger RNA
NEC: non-exposed cells
NS3: Non-structural protein 3
NS5A: Non-structural protein 5A
P(-): Pellets from uninfected U937 macrophages
P(+): Pellets from DENV-2 infected U937 macrophages
PBS: Phosphate buffered saline
PFA: Paraformaldehide
PFU: Plaque forming units
prM: pre-membrane viral protein
PQ: Plaque Assay
PS: Penicillin/Streptomycin
PVDF: Polyvinylidene fluoride
TEER: Transendothelial electrical resistance
TEM: Transmission electronic microscopy
TGF-β: Transforming growth factor beta
UC: ultracentrifugation
WB: Western Blot
