# Supplementary material for: Extracellular vesicles of U937 macrophage cell line infected with DENV-2 induce activation in endothelial cells EA.hy926
Source: PLoS One. 2020 Jan 7;15(1):e0227030. doi: 10.1371/journal.pone.0227030 (PMC6946137; doi:10.1371/journal.pone.0227030)
Supplement: S1 File — A pdf file that contains all the raw images of the blots presented in this paper. https://dataverse.harvard.edu/dataset.xhtml?persistentId=doi:10.7910/DVN/1Y17SG. (PDF) [file pone.0227030.s001.pdf]

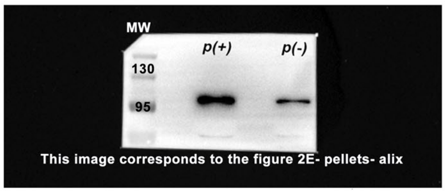

This image corresponds to the figure 2E- pellets- alix

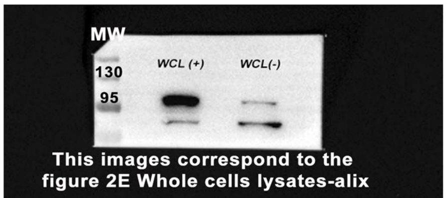

This images correspond to the figure 2E Whole cells lysates-alix

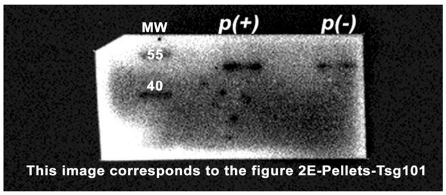

This image corresponds to the figure 2E-Pellets-Tsg101

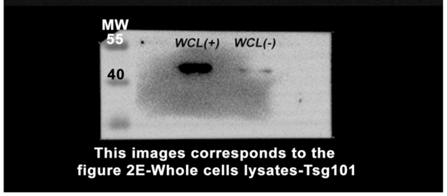

This images corresponds to the figure 2E-Whole cells lysates-Tsg101

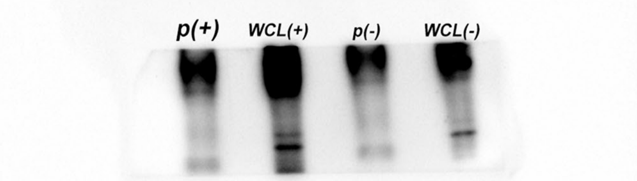

This images corresponds to the figure 2E pellets and whole cell lysates-CD63

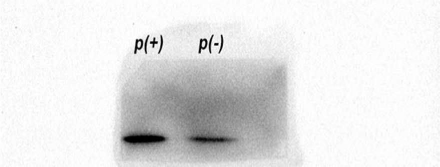

This image correspond to the figure 2E-pellets-H3

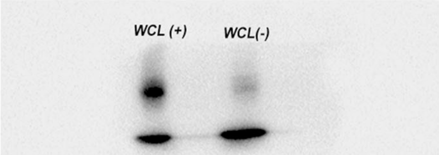

This images correspond to the figure 2E-Whole cells lysates-H3

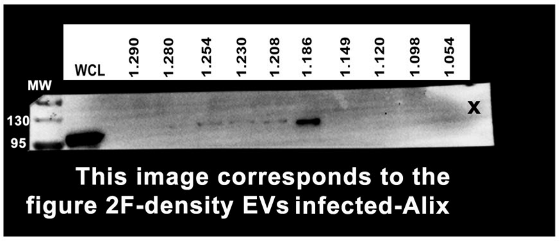

This image corresponds to the figure 2F-density EVs infected-Alix

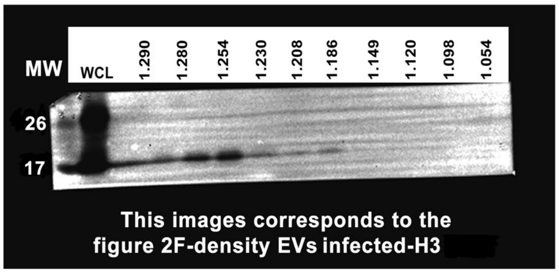

This images corresponds to the figure 2F-density EVs infected-H3

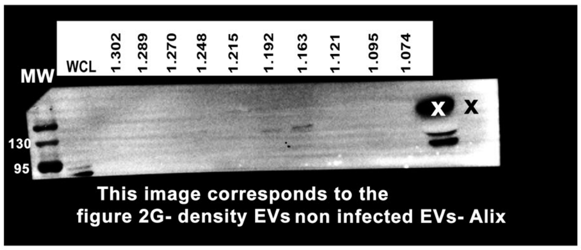

This image corresponds to the figure 2G- density EVs non infected EVs- Alix

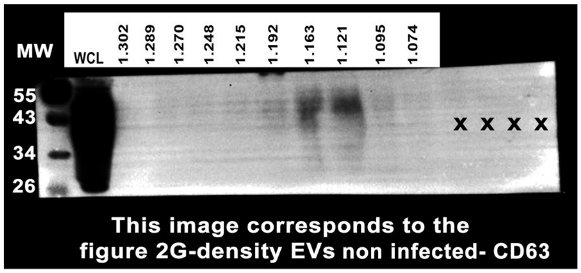

This image corresponds to the figure 2G-density EVs non infected- CD63

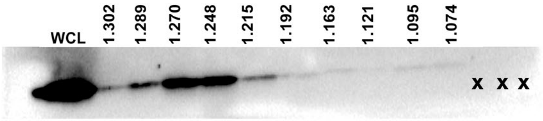

This image corresponds to the figure 2G density EVs non infected-H3

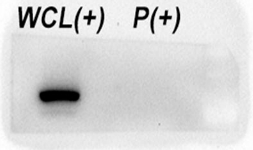

This image corresponds to the figure 3A-
whole cell lysates and pellets of infected cells -NS5

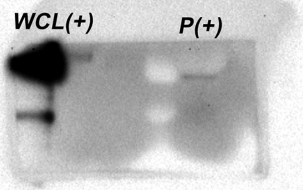

This mages corresponds to the figure 3A-
Whole cells lysates and pellets of infected cells -NS3

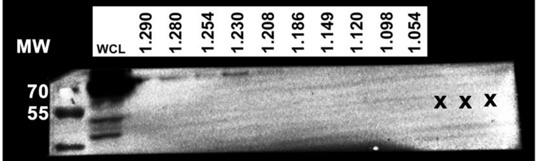

This images corresponds to the figure 3B-
density EVs infected cells-NS3

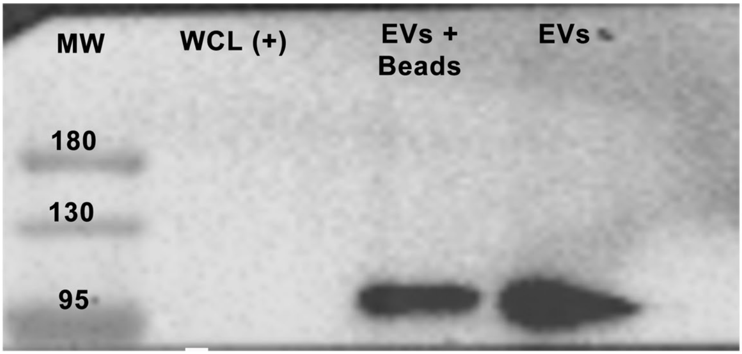

This image corresponds to the figure supple 1- EVs IP- Alix

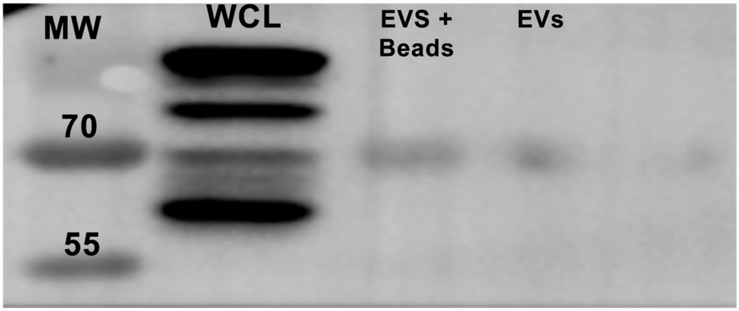

This image corresponds to the figure supple 1- EVs IP- NS3

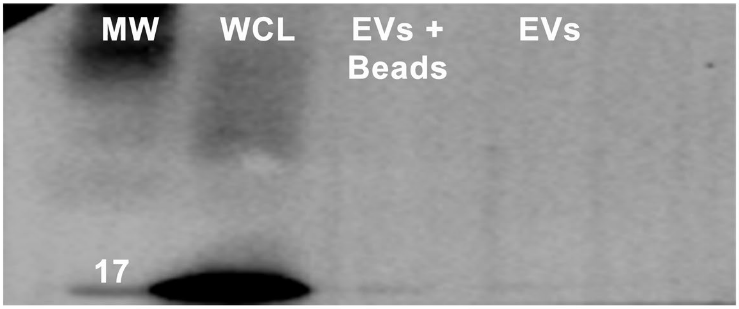

This image corresponds to the figure supple 1- EVs IP- h3
