# Supplementary figures and images for: Extracellular vesicles of U937 macrophage cell line infected with DENV-2 induce activation in endothelial cells EA.hy926
Source: PLoS One. 2020 Jan 7;15(1):e0227030. doi: 10.1371/journal.pone.0227030 (PMC6946137; doi:10.1371/journal.pone.0227030)

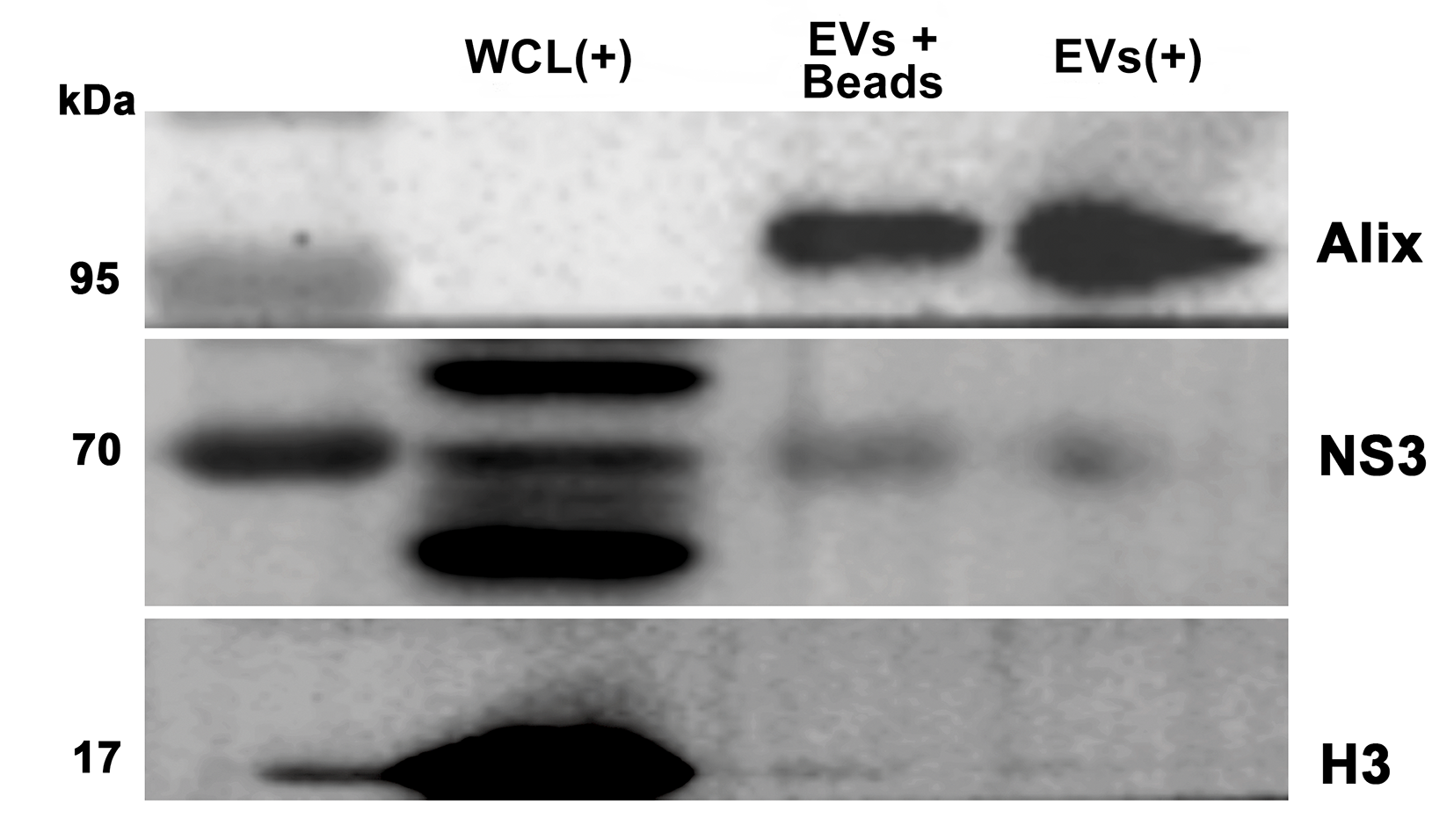

Supplement: S1 Fig — Extracellular vesicles obtained from DENV infected and non-infected macrophages were pelleted by ultracentrifugation, processed by immunoprecipitation (IP) and then separated by electrophoresis. Purified vesicles from infected U937 cells (EVs (+)) were positive to Alix and NS3 but slightly to H3. Control conditions were, infected whole cells lysate (WCL (+)), and the EVs bound to CD63 beads (EV + beads). (TIF) [file pone.0227030.s002.tif]

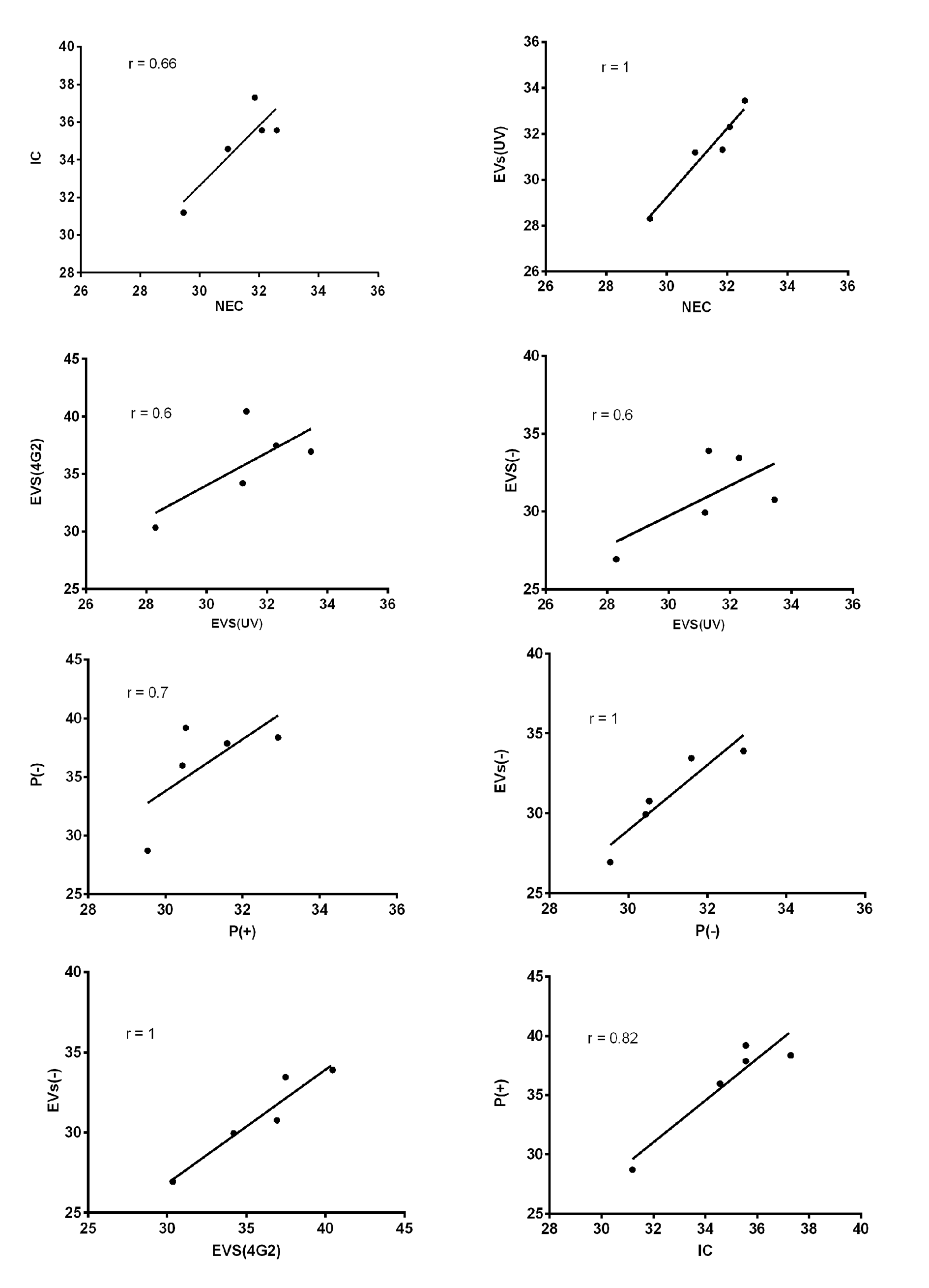

Supplement: S2 Fig — Scatter plots with lineal regressions correlating the obtained TEER data in every evaluated condition. Graphs show for every comparable group of data (NEC vs IC; P (-) vs P (+); NEC vs EVs (+) UV vs EVs (+) 4G2 vs EVs (-); and P (+) vs IC) as well as the correlation coefficient (r) for them. (TIF) [file pone.0227030.s003.tif]

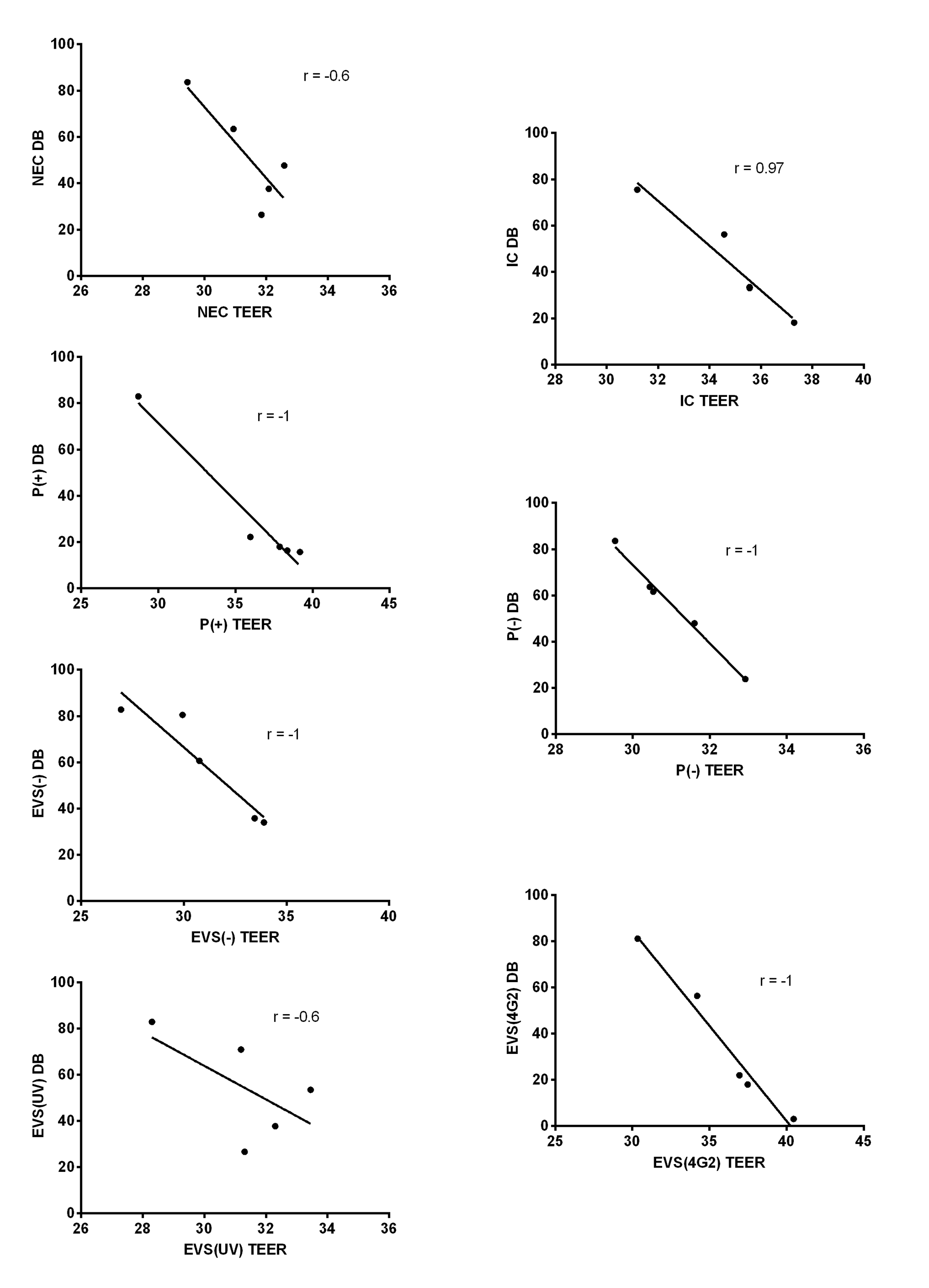

Supplement: S3 Fig — Scatter plots with lineal regressions correlating the obtained results for TEER and permeability for all evaluated conditions showing the correlation coefficient (r) for them. (TIF) [file pone.0227030.s004.tif]
